# Single-cell metabolome and RNA-seq multiplexing on single plant cells

**DOI:** 10.1073/pnas.2512828122

**Published:** 2025-10-24

**Authors:** Moonyoung Kang, Anh Hai Vu, Abbie L. Casper, Rinho Kim, Jens Wurlitzer, Sarah Heinicke, Assa Yeroslaviz, Lorenzo Caputi, Sarah E. O’Connor

**Affiliations:** ^a^Department of Natural Product Biosynthesis, Max Planck Institute for Chemical Ecology, Jena 07745, Germany; ^b^Next Generation Sequencing and Bioinformatics Core Facility, Max Planck Institute for Biochemistry, München 82152, Germany

**Keywords:** single-cell-omics multiplexing, single-cell metabolome, single-cell transcriptome, plant natural products, *Catharanthus roseus*

## Abstract

Plants produce valuable natural products used for a wide variety of industrial applications. Thus, there is enormous interest in elucidating the biosynthetic pathways that are responsible for the production of these compounds. Identification of the genes that comprise these biosynthetic pathways has been enabled by gene-to-metabolite networks that are generated from transcriptomic and metabolomic datasets. Recent advances in both single-cell RNA-seq (scRNA-seq) and single-cell mass spectrometry metabolomics (scMS) have enabled the measurement of either gene expression or metabolite levels in individual cells. However, these datasets can only be used to indirectly correlate gene expression levels with metabolite concentrations at the single-cell level. In this proof-of-concept study, performed on cells derived from the leaves of the medicinal plant *Catharanthus roseus*, we demonstrate that both scRNA-seq and scMS can be applied to the same plant cell, thereby enabling direct comparisons between gene expression and metabolite levels. Protoplasts are sorted into 96-well plates using a microfluidics-based robot and then lysed under conditions that are suitable for both scMS and SMART-seq single-cell protocols. This multiplexing approach reveals both qualitative and quantitative correlations between metabolite levels and biosynthetic gene expression in individual cells. This integrated approach sheds light on the underlying processes driving complex plant biosynthesis.

## Significance

Plants produce valuable metabolites through the action of complex biosynthetic pathways, metabolic processes that are typically composed of many genes. Advances in single-cell omics now allow measurement of either gene expression or metabolite levels within individual cells. Here, we demonstrate that these two approaches can be applied to the same single cell. This enables the generation of matched gene–metabolite datasets, allowing rigorous correlation analyses at single-cell resolution. Such integration could facilitate the discovery of the genes involved in metabolite production, which in turn may improve access to these valuable molecules.

Plants produce an extraordinary array of complex natural products. Since these molecules have important applications in pharmaceutical, agricultural, and nutritional sectors, there is enormous interest in elucidating the biosynthetic pathways that are responsible for the production of these plant-derived compounds. Identification of the genes that comprise these biosynthetic pathways has been greatly facilitated by the availability of transcriptomic and metabolomic datasets. These data are used to construct gene-to-metabolite networks that allow correlation of gene expression with metabolite levels. Such correlation analyses have led to the discovery of many biosynthetic genes, transporters, and regulatory elements from plant pathways (1–6).

Single-cell transcriptomic (scRNA-seq) approaches have enabled a step-change in the resolution to which gene expression profiles can be measured (7, 8). scRNA-seq is now well-adapted for a number of plant systems, and, since plant biosynthetic genes of specialized metabolism are typically localized to only a few specific cell types, scRNA-seq is particularly well-suited for analysis of natural product pathways (9–11). However, generation of accurate gene-to-metabolite correlations at the single-cell level requires, in addition to scRNA-seq datasets, corresponding single-cell mass spectrometry (scMS) data. We recently reported a scMS method for analysis of single plant protoplasts and subsequently demonstrated how the resulting scMS data could be interpreted alongside scRNA-seq to facilitate the discovery of biosynthetic genes (10, 12). However, correlations between these two distinct datasets could only be made indirectly as the generation of gene-to-metabolite networks using these data was not possible.

We envisioned that rigorous gene-to-metabolite correlations could be achieved through a multiplexed approach in which both scMS and scRNA-seq data are obtained from the same individual plant cell. Here, we report a method by which a single plant protoplast can be analyzed by RNA-seq and metabolomics. Using *Catharanthus roseus*, a medicinal plant known for producing an array of terpenes, alkaloids, and phenylpropanoid natural products as a proof-of-concept (13), we demonstrate qualitative and quantitative correlations between metabolite levels and biosynthetic gene expression at the single-cell level. These datasets show how the intermediates and end-products in the pathway of the alkaloid anhydrovinblastine, a precursor to the anticancer agent vinblastine, are located relative to the biosynthetic genes. Overall, this integrated method offers a powerful tool for understanding the spatial organization of metabolite biosynthesis in plants and may accelerate the discovery of genes involved in specialized biosynthetic pathways.

## Results

### Bulk Analysis of Tissue and Protoplasts From *C. roseus* Young Leaves.

Since our scMS approach necessarily relies on the use of protoplasts (10, 12, 14), we first established the degree to which tissue dissociation (“protoplasting”) impacts gene expression and metabolite profiles. To this end, we performed metabolomic and transcriptomic analyses using intact leaf tissue and compared these datasets to those generated from an aliquot of protoplasts taken at two time points: 2.5 h (corresponding to the time required to generate protoplasts from leaf dissection) and 4.0 h (an additional 90 min incubation, exceeding the duration required for cell picking).

Extracted ion chromatograms of four major metabolites—catharanthine, secologanin, serpentine, and vindoline—showed that the levels of these compounds in protoplast preparations at both the 2.5 h and 4.0 h time points were comparable to the levels measured in intact leaf tissue (*SI Appendix*, Fig. S1). In contrast, global transcriptome comparisons by Spearman correlation revealed some changes in gene expression following protoplasting (coefficient = 0.85) (*SI Appendix*, Fig. S2). The extent of change that we observed was comparable to that observed in other studies (15, 16). The correlation of gene expression between the 2.5 h and 4.0 h timepoints was high (coefficient > 0.96), suggesting that no major transcriptomic changes occur in the protoplasts over time (*SI Appendix*, Figs. S2 and S3).

GO term enrichment analysis revealed several transcriptional regulators were the major genes upregulated during protoplasting (*SI Appendix*, Fig. S4). Closer inspection showed that many genes involved in iridoid biosynthesis, localized to Internal Phloem-Associated Parenchyma (IPAP) cells, as well as late-stage alkaloid biosynthetic genes that are localized to idioblast cells, were downregulated following tissue dissociation (*SI Appendix*, Fig. S5). These changes in gene expression could reflect a physiological stress response to protoplasting. Alternatively, since the biosynthetic genes expressed in the highly abundant epidermal cells were comparatively unaffected, it is possible that the protoplasting process may not capture all cell types with equal efficiency.

Finally, to determine whether there was bias in the cell picking process, we calculated the correlation between the transcriptome obtained for the bulk protoplasts at the 2.5 h time point with the combined transcriptomes of all single cells analyzed in this study (“pseudobulk”). These two transcriptomes were well correlated, indicating that no significant bias was generated by the cell picking process and the cDNA library preparation (*SI Appendix*, Fig. S6).

### scRNA-seq–scMS Multiplexing Workflow and Data Acquisition.

**Fig. 1.**
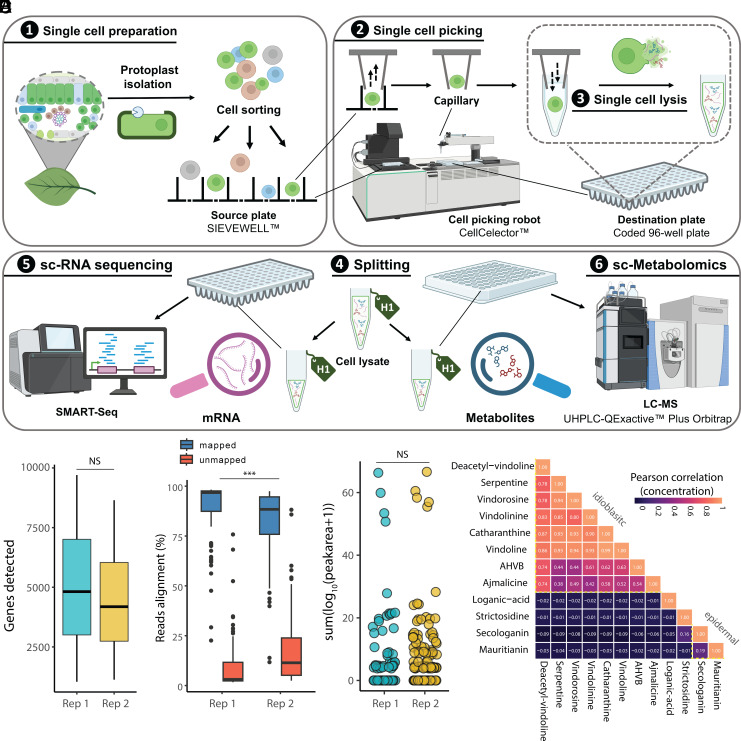
Multiplexing of single plant protoplasts. (*A*) Workflow for simultaneous single-cell transcriptomics and metabolomics of plant protoplasts. (*B*) Number of genes detected in single cells. (*C*) Sequencing read alignment ratio to genome. (*D*) Number of metabolites detected in single cells. Statistical differences between the two groups were calculated by performing two-sided Kolmogorov–Smirnov (KS) tests (***P < 0.01; NS, not significant). For boxplots, the box represents the interquartile range (IQR), and the whiskers indicate the highest and lowest points within 1.5× IQR. (*E*) Summary of metabolomics profile, performed as described in a previous study (12). Pearson correlation coefficients between concentrations of 12 analytes are visualized based on color scale.

The scMS method used here relies on sorting plant protoplasts on a SIEVEWELL™ chip (10, 12). After being trapped into an individual well of the chip, each protoplast is picked using a robot system (CellCelector™ Flex), and transferred into a small volume of water in a microtiter plate. The resulting osmotic shock causes immediate lysis. After addition of solvent and internal standard, an aliquot of this solution is analyzed using a standard UPLC-MS instrument. Because this method includes a chromatographic step, metabolites can be structurally identified by comparison of retention time and MS/MS fragmentation with authentic standards. Additionally, using calibration curves for these standards, along with cell diameter measurements obtained from images captured during the picking process, metabolite concentrations can be calculated for each cell. We reasoned that this scMS workflow could be adapted for multiplexing with a plate-based scRNA-seq approach (17). To preserve RNA integrity, protoplasts were dispensed into 8 µL of RNase-free water containing RNase inhibitor. Following lysis, each well’s contents were split: Half was used to generate a replica plate for RNA-seq, stored at –80 °C prior to cDNA library preparation using the SMART-seq plate-based protocol, while the other half was used for UPLC-MS analysis (Fig. 1*A*).

Protoplasts were isolated from young leaves of the medicinal plant *C. roseus*, which are an active site of alkaloid biosynthesis (18). To ensure sufficient number of cells for the experiment, a total of four 96-well plates were collected on separate days. Two plates were prepared each time from freshly isolated protoplasts derived from leaves of two individual plants. Image analysis from the cell picking process confirmed that 289 cells were successfully picked, while 12 wells contained cell doublets and 95 wells were empty. The lysates from these 289 cells were subjected to both transcriptomic and metabolomic analysis. Following initial quality assessment of the scRNA-seq data, cells with fewer than 1,000 detected genes were excluded from the dataset, resulting in a total of 193 (66%) retained for further analysis. Across the entire dataset, a total of 21,258 genes were identified, with each cell expressing an average of 5,000 genes (Fig. 1*B*) with a mapping rate onto the genome (cro_v3) greater than 80% (Fig. 1*C*) (10). No significant batch variation was observed when we combined the data from the cells collected on the two different days (*SI Appendix*, Fig. S7). We note that the use of a plate-based scRNA-seq protocol enabled us to detect a substantially greater number of genes per cell compared to droplet-based approaches (two- to threefold increase in sequencing depth) (10, 19), yielding deep transcriptomic profiles well suited for gene mining.

**Scheme 1.**
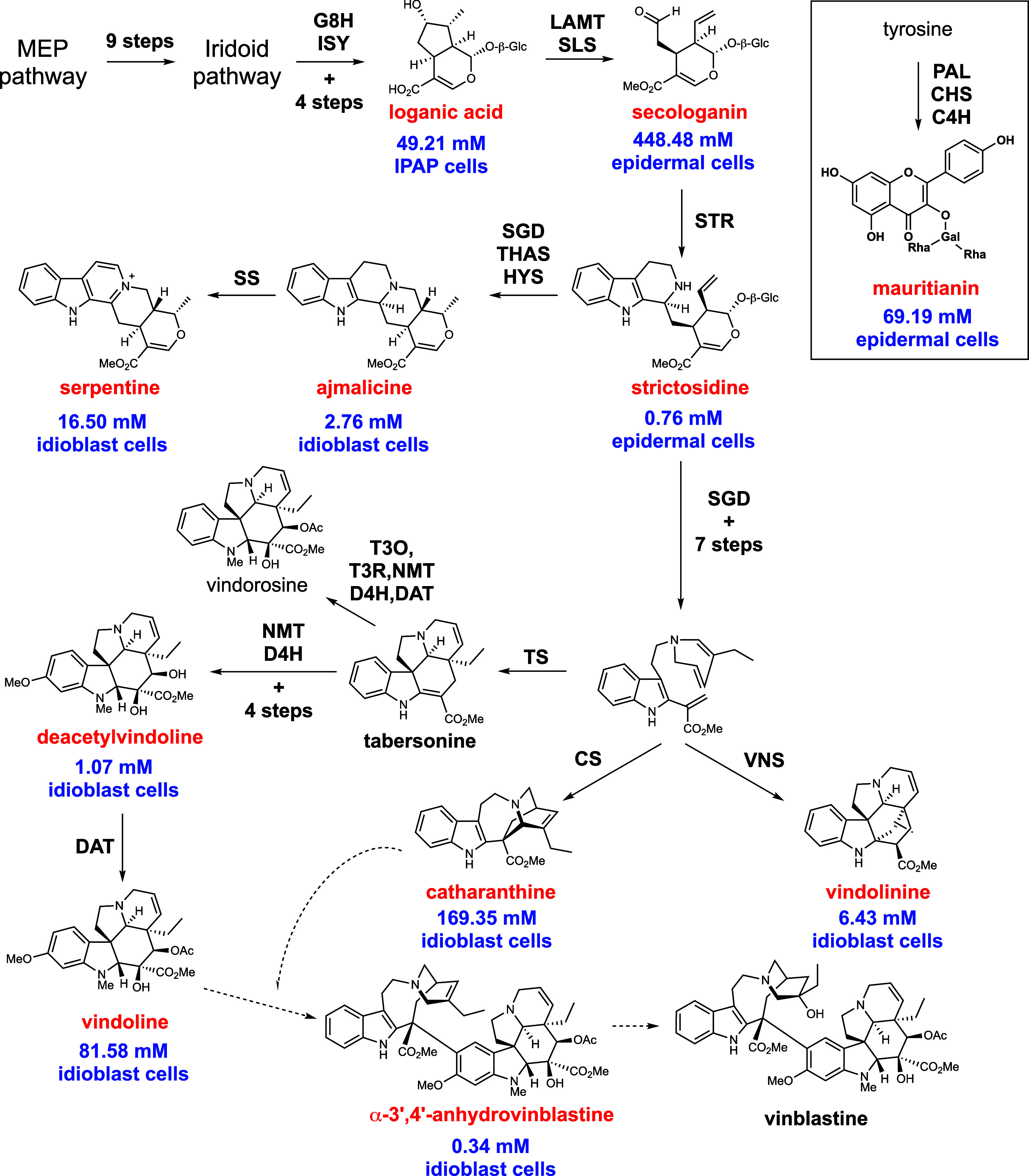
Pathway to metabolites of *Catharanthus roseus.* Key metabolites targeted in this study are highlighted in red text. The mean intracellular concentrations measured in this study are reported in blue text. Selected biosynthetic genes are indicated in black. Dashed lines indicate that biosynthetic genes for these steps are unconfirmed. Mauritianin, which is biosynthetically unrelated to the other metabolites targeted, is shown in the inset box. A complete biosynthetic pathway including the full name of the enzymes is shown in *SI Appendix*, Scheme 1.

We next analyzed the scMS data from the same set of 193 single cells. Our analytical workflow allows the acquisition of both untargeted and targeted data in the same experiment. Targeted mass spectrometry was employed to quantify 14 metabolites of interest, for which authentic standards are available (*SI Appendix*, Table S1). For all these metabolites, external calibration parameters and limit of quantification (LOQ) were calculated and are reported in *SI Appendix*, Table S2. Under these conditions, intracellular concentrations were successfully quantified for 12 of the 14 metabolites (*SI Appendix*, Tables S2 and S3). Notably, several key intermediates in the anhydrovinblastine biosynthetic pathway, including the iridoid loganic acid, the seco-iridoid secologanin, and the alkaloids catharanthine and vindoline, were detected at high concentrations, reaching millimolar levels in some cells (Scheme 1). Anhydrovinblastine itself accumulated in micromolar concentrations, while vinblastine, typically present in much lower levels, was not observed in this limited sample set (*SI Appendix*, Table S3). Additional intermediates in the pathway, such as strictosidine and deacetylvindoline, were observed at low micromolar concentrations (*SI Appendix*, Table S3). We also quantified several related alkaloids—vindorosine, vindolinine, ajmalicine, and serpentine—which are biosynthetically related to, but not direct intermediates of, anhydrovinblastine (Scheme 1). In addition to these alkaloids, we quantified the flavonoid mauritianin, a kaempferol diglycoside, which is one of the major flavonoids found in *C. roseus* leaf. All metabolites were quantified across the 193 cells analyzed (*SI Appendix*, Figs. S8 and S9). To assess whether metabolites are released or actively exported into the medium, we also analyzed wells in which no cells had been dispensed. Only trace amounts of the most abundant metabolites were occasionally detected, suggesting minimal leakage or export of metabolites from viable protoplasts (*SI Appendix*, Fig. S10). Finally, comparison of data collected on two separate days showed high consistency between biological replicates and aligned well with previously reported scMS data for this species (*SI Appendix*, Tables S3 and S4).

Correlation analysis of the scMS dataset revealed that all nitrogen-containing alkaloids, with the exception of strictosidine, consistently co-occurred within the same cells (Fig. 1*E*). Based on the presence of vindoline, these cells were tentatively identified as idioblast cells (10, 12, 20). Strictosidine is rarely detected in leaf protoplasts, but when present, it co-occurs with its precursor secologanin (correlation r > 0) (Fig. 1*E*). Secologanin is predicted to localize primarily in the epidermal cell (21). Although flavonoids in *C. roseus* are also predicted to be localized to epidermal cells (22), mauritianin was detected only in a subset of cells that also accumulated secologanin (*SI Appendix*, Fig. S11*B*) (12). Loganic acid, which is synthesized in internal phloem-associated parenchyma (IPAP) cells (23), was used as a marker to tentatively classify loganic acid–accumulating cells as IPAP cell types.

Untargeted metabolomic analysis yielded a global dataset of 2,331 features with assigned elemental compositions. After further filtering, 840 robust features remained, which enabled clustering of the protoplasts into four distinct cell types (*SI Appendix*, Fig. S12). Each of the 12 metabolites that were subjected to quantification was also detected in the untargeted dataset. These positively identified metabolites were used as markers for the cell clusters generated from the untargeted analysis.

### Cell Clustering Based On Gene Expression and Metabolite Accumulation.

A uniform manifold approximation and projection (UMAP) was generated from the scRNA-seq data (RNA-UMAP), and cell types were annotated based on previously validated marker genes (19, 24–26). The gene *NLTP2* was used as a marker gene for epidermis, while *CB21*, known not to be expressed in epidermal cells, was used to validate this assignment (19) (*SI Appendix*, Fig. S11*A*). *DAT* and *D4H* transcripts served as markers for idioblasts. Cells were annotated as IPAP when either *G8H* or *ISY* transcripts, which are reported to be highly specific to this cell type, were detected (25, 26). In the RNA-UMAP, cells expressing epidermal and idioblast markers formed well-defined clusters (*SI Appendix*, Fig. S11*A*). In contrast, no distinct cluster corresponding to IPAP cells was observed. *ISY* was expressed at low levels in only two cells, while *G8H* was detected in just six cells, all located outside the idioblast and epidermal clusters (*SI Appendix*, Fig. S14). We assigned these cells as IPAP type, even though *G8H* and *ISY* were expressed at low levels. However, in actuality, this sample of protoplasts may not have included IPAP cells, which are known to be a rare cell type, comprising <4% of the total leaf cell population, based on previous studies (27). The close proximity to the internal phloem, often near vascular tissues, might have prevented efficient enzymatic dissociation under the conditions used in this study.

We next projected cell type–specific metabolites onto this RNA-UMAP (*SI Appendix*, Fig. S15*A*). As expected, secologanin localized to the same region as the epidermal marker gene (*SI Appendix*, Figs. S11*A* and S15A), consistent with its predicted epidermal origin. Similarly, the flavonoid mauritianin also mapped to the epidermal region. In contrast, alkaloids, e.g., serpentine and anhydrovinblastine (AHVB), correlated to the idioblast region of the RNA-UMAP, in line with the known localization of these compounds (*SI Appendix*, Figs. S11*A* and S15A). Loganic acid, which is predicted to be synthesized in IPAP cells, showed no correlation with cells expressing IPAP marker genes (*SI Appendix*, Figs. S15 and S16*B*). This is consistent with our earlier observation that a distinct IPAP cell cluster was not evident on the RNA-UMAP (*SI Appendix*, Fig. S11*A*). Given that loganic acid must be transported from IPAP cells to epidermal cells (21, 23), where it is converted to secologanin, we initially hypothesized that its presence might coincide with epidermal cell markers. However, loganic acid did not correlate with the epidermal cell either. Instead, the seven cells in which loganic acid was detected were primarily located in the parenchyma cell region of the RNA-UMAP (*SI Appendix*, Figs. S11*A* and S15). It is possible that the high concentrations of loganic acid observed (average intracellular concentration of ca. 40 mM) represent molecules that have been exported from the IPAP cells and stored in a distinct, yet uncharacterized, cell type. The cell type(s) in which loganic acid accumulates could not be confidently annotated due to the limited availability of specific gene markers for plant cell types.

**Fig. 2.**
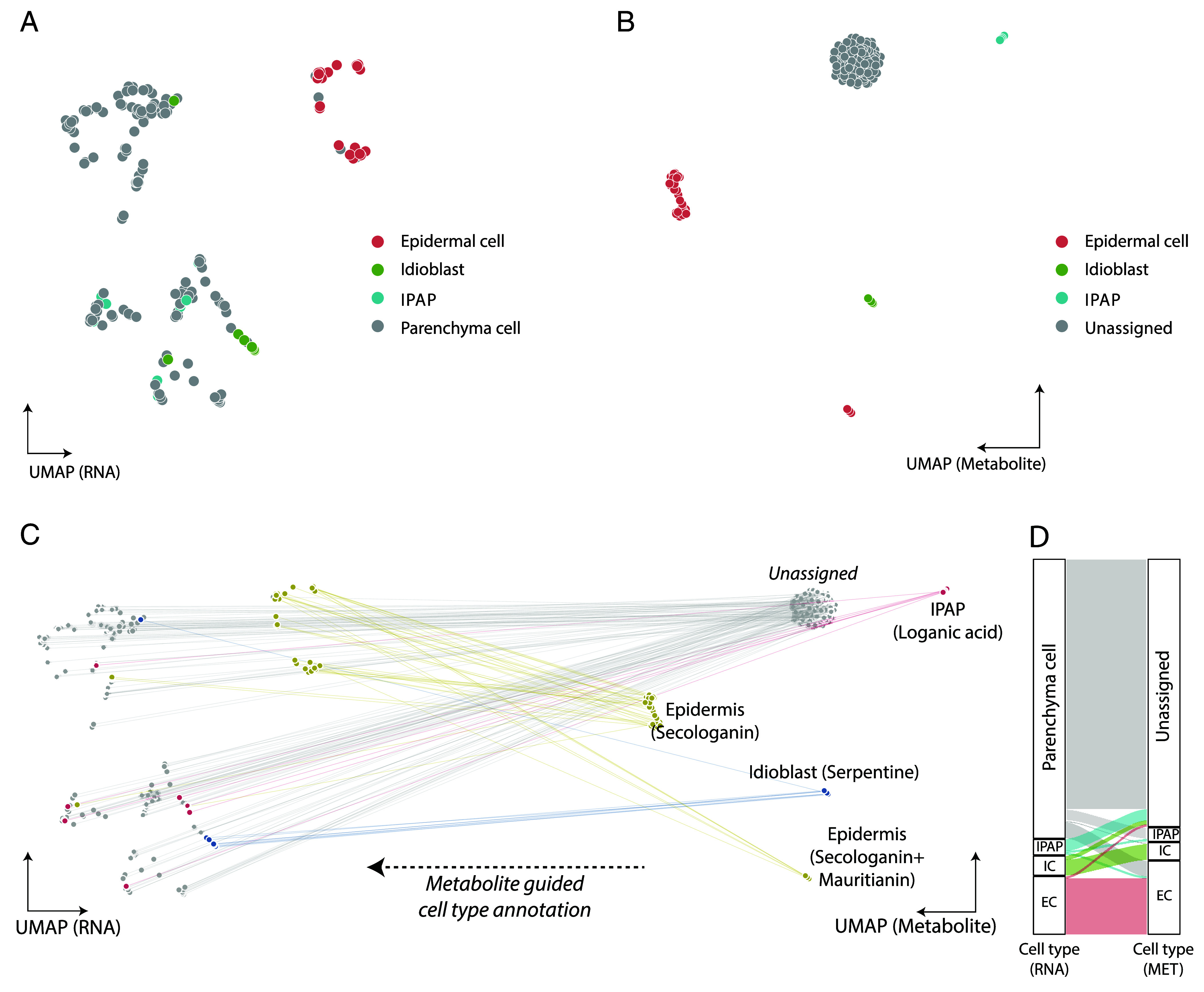
UMAP showing clustering of cell types by gene expression and metabolite expression. (*A*) Marker gene-guided cell type annotation in RNA-UMAP plot. (*B*) Targeted analyte-guided cell type annotation in MET-UMAP plot. (*C*) These two different UMAP plots were connected based on their corresponding well identities, where cells are colored with annotations based on representative metabolite accumulation (serpentine, secologanin, mauritianin, loganic acid). (*D*) Sankey plot showing similarities and differences when cells are annotated with gene markers or metabolites. The height of the plot is proportional to the number of cells. The *Left* side represents the marker gene-based cell type annotation, and the *Right* side represents metabolite-based annotation.

To further investigate metabolite distribution across cell types, we generated a UMAP based on the 12 quantified metabolites rather than gene expression data (targeted-MET-UMAP, Fig. 2*C*) and a UMAP based on untargeted metabolomics data (untargeted-UMAP, *SI Appendix*, Fig. S13). This analysis revealed distinct clusters of cells enriched in specific compounds, including alkaloids (e.g., serpentine, vindoline, anhydrovinblastine), secologanin, mauritianin, or loganic acid (Fig. 2*B* and *SI Appendix*, Fig. S11*B*). We then compared the RNA-UMAP and targeted-MET UMAP to assess the spatial correlation of selected gene–metabolite pairs (Fig. 2*C*). Notably, 8 out of 9 (89%) cells that accumulate serpentine also expressed the idioblast cell markers *D4H* and *DAT*. Similarly, secologanin showed strong correlation with the epidermal marker *NLTP2*, with 29 out of 38 (76%) cells that accumulate secologanin also expressing this marker (Fig. 2*D* and *SI Appendix*, Table S4). Remarkably, the targeted-MET-UMAP revealed two distinct clusters within the epidermal population: one accumulating secologanin alone and the other accumulating both secologanin and the flavonoid mauritianin (Fig. 2*C* and *SI Appendix*, Fig. S11*B*). Cells in both clusters corresponded to *NLTP2*-expressing cells in the RNA-UMAP, suggesting the existence of two metabolically distinct cell subtypes. Transcriptomic comparison of these two epidermal subpopulations revealed several differentially expressed genes, including upregulation of *KCS* and lipid transfer proteins in the mauritianin-accumulating cells (*SI Appendix*, Figs. S19 and S20). However, expression levels of predicted flavonoid biosynthetic genes (e.g., *PAL, CHS, C4H*) did not differ significantly between the two groups, suggesting that the genetic basis underlying their metabolic differentiation may be more complex than anticipated.

### Quantification of Gene–Metabolite Correlations.

**Fig. 3.**
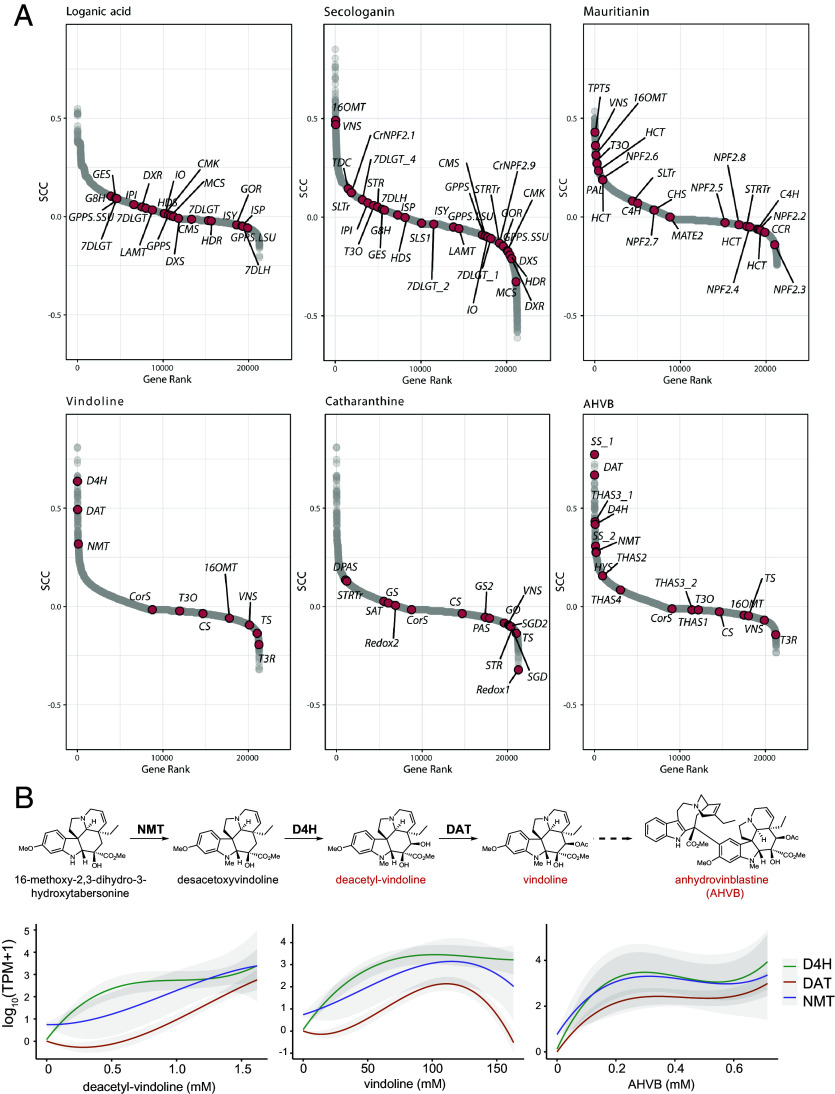
Quantitative gene to metabolite correlation. (*A*) Spearman correlations of all genes were plotted according to their correlation rank with each of the listed compounds. (*B*) Trend lines (local regression with 95% CI) of NMT, D4H, and DAT expression levels (TPM) are plotted according to ascending concentration of each listed compound.

Since both gene expression levels and selected metabolite concentrations were quantified in this study, we were able to assess gene–metabolite relationships using Spearman correlation analysis (Fig. 3*A*). Specifically, we calculated the correlation between the occurrence of specific metabolites and the expression levels of all detected genes across the dataset. For several metabolites, we observed strong correlations with the expression of known biosynthetic genes. For example, vindoline showed high correlation with the expression of the genes *D4H*, *DAT,* and *NMT*—the final genes in its biosynthetic pathway (Scheme 1). This strong correlation is consistent with the fact that vindoline is stored in the same cell type in which it is synthesized. Similarly, anhydrovinblastine, a downstream, derivatized product of vindoline, that is also localized in the idioblasts, showed strong correlation with *D4H, DAT,* and other idioblast-enriched genes.

In contrast, some biosynthetic genes showed weak or no correlation with alkaloid accumulation, likely due to extensive transport between cell types. While alkaloids are ultimately stored in idioblasts (*SI Appendix*, Fig. S17), many upstream steps in their biosynthetic pathway take place in epidermal cells (*SI Appendix*, Figs. S16*C* and S18). For instance, *T3O* and *T3R*, which function early in vindoline biosynthesis and are localized to the epidermis, did not correlate with vindoline presence (Fig. 3*A*). Similarly, catharanthine is synthesized in epidermal cells but subsequently transported to idioblasts for storage. As a result, we observed no meaningful correlation between catharanthine and the expression of its biosynthetic genes (e.g., *DPAS, PAS, CS)* (Fig. 3*A*).

We also examined correlations for secologanin and mauritianin, two metabolites that are expected to be both synthesized and retained within epidermal cells. While the correlation with specific biosynthetic genes was modest (e.g., *LAMT* for secologanin, *C4H* for mauritianin), both metabolites showed strong correlation with a range of genes known to be expressed in epidermal cells, such as *16OMT* and *VNS*. Thus, although direct correlations with individual biosynthetic genes were limited, these metabolites aligned well with markers for the correct cell type. In contrast, the iridoid loganic acid, which is synthesized in IPAP cells and then transported to epidermal cells for conversion into secologanin, did not show significant correlation with either IPAP- or epidermis-specific genes, as discussed above (Figs. 2*D* and 3*A*). This reinforces the hypothesis that the large pools of loganic acid observed by scMS likely reflect transport to a third, unidentified cell type where it is stored or transported through.

We next examined the relationship between absolute metabolite concentrations and the expression levels of specific biosynthetic genes (Fig. 3*B*). As a proof of concept, we focused on the idioblast-localized metabolites deacetylvindoline, vindoline and anhydrovinblastine, and their corresponding biosynthetic genes *NMT*, *D4H,* and *DAT*, as these gene–metabolite pairs showed strong qualitative correlations. We observed that the expression of these biosynthetic genes generally increased in parallel with rising alkaloid concentrations, reaching peak expression at approximately 100 mM metabolite level (Fig. 3*B*). Beyond this point, gene expression levels began to decline slightly. We speculate that this pattern may reflect a developmental trajectory of idioblasts, transitioning from active biosynthesis to storage phase, in which mature cells accumulate high alkaloid concentrations while exhibiting reduced gene expression. Alternatively, these high levels of alkaloid may lead to feedback inhibition of gene transcription. However, the relatively small number of idioblasts captured in this dataset limits our ability to test these hypotheses with statistical confidence.

### Relationship of Transporters and Metabolites.

**Fig. 4.**
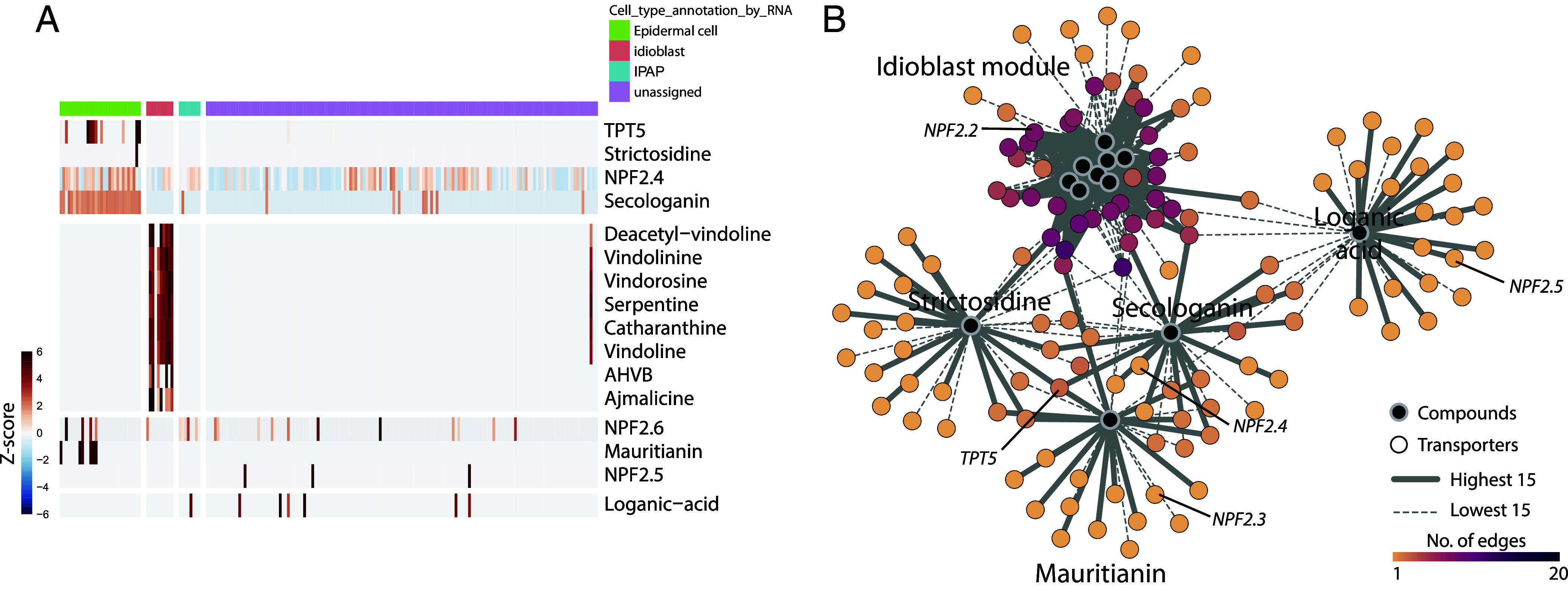
Correlation between transporter gene expression and metabolite occurrence in different cell types. (*A*) Heatmap showing coexistence of known intercellular transporter–metabolite pairs. Z-scores of each gene expression and metabolite accumulation in a single cell were colored and hierarchically clustered. (*B*) Expected network of metabolites and putative importers/exporters based on correlation. Transporters with the *Top* 15 highest and *Bottom* 15 lowest correlation values are linked to metabolites in the network. The number of connections for each transporter is represented by a color scale. Transporters are annotated if they were studied or mentioned in the literature.

Given the extensive intercellular transport involved in these metabolic pathways, we also assessed the correlation between metabolites and expression of the genes encoding previously identified transporters (Fig. 4*A*). NPF2.4, 2.5, and 2.6 were shown to transport loganic acid, loganin, and secologanin in an in vitro assay (28). Our data suggest that NPF2.4 is the physiologically relevant transporter that imports iridoids into the epidermal cells, as its expression profile closely mirrors the accumulation of secologanin in this cell type (Fig. 4*A*). TPT2, which has been reported to export catharanthine from epidermal cells to cuticle layer (29), was not detected in any of the 193 cells analyzed, suggesting it may not play a significant role in catharanthine transport in planta, or that it may not be expressed in young leaves. However, a homologous gene, TPT5, was expressed in a subset of epidermal cells, though its function is unknown (29). Although these are the only intercellular transporters characterized to date in *C. roseus*, the integrated transcriptomic and metabolomic dataset presented here provides a valuable resource for identifying additional candidate transporters involved in specialized metabolites trafficking.

## Discussion

Here, we demonstrate that small molecule mass spectrometry and RNA-seq can be simultaneously applied to a single plant protoplast. Multiplexing approaches that integrate metabolomics at the single-cell level are not widely reported, due to the challenges associated with observing and annotating small molecules in single cells. To date, the only other reported single-cell multiplexing approach involving metabolomics employed a nano capillary to manually sample just fifteen mammalian cells (30). While that study represents a technically rigorous and elegant approach, the method presented here is specifically adapted for plant cells, offers significantly higher throughput, and enables accurate quantification of individual metabolites.

Both scMS and scRNA-seq provide valuable insights into cellular metabolism, each with its own limitations. Single-cell metabolomics reveals the identity and quantities of selected molecules in cells, but in the absence of gene expression data, the biological interpretation of these measurements remains limited. Conversely, single-cell RNA-seq data provide information about the location of biosynthetic genes, but cannot directly link genes to their corresponding metabolites. By simultaneously profiling the transcriptome and metabolome of a single cell, we are able to directly associate cell type markers and biosynthetic genes with the presence of specific metabolites. This integrated dataset therefore distinguishes metabolites that remain localized at the site of synthesis with those that are transported elsewhere. For example, we observed that pools of loganic acid were absent from idioblasts and epidermal cells, indicating that this iridoid pathway intermediate is transported to, and stored in, a different and unexpected cell type. Thus, this integrated approach offers a promising strategy to infer which metabolites are translocated and to link these translocation processes with cell type–specific transporters. In contrast, late-stage alkaloid intermediates are both synthesized and retained in idioblast cells, as evidenced by strong quantitative correlations between gene expression and metabolite concentration. Importantly, the construction of metabolite–gene networks provides a framework to track gene expression dynamics along metabolite concentration gradients at single-cell resolution (Fig. 3). However, we note that, due to differences in translation efficiency and protein turnover, transcript abundance does not always correlate with protein levels (31, 32). This inherent limitation likely influences the strength of the gene–metabolite correlations we report.

As with all analytical methods, the limitations inherent within the approach must be taken into account when interpreting the resulting data. First, this method is restricted to cells that can be successfully isolated as protoplasts. While earlier studies demonstrated that the genes involved in MIA biosynthesis are still expressed in cell type–specific manner after protoplast formation (10), certain biological processes are inevitably perturbed by cell wall digestion and thus may not be faithfully captured with this approach. Additionally, we speculate that not all cell types may be equally well captured by the protoplasting process (19). Recently, transcriptome fixation using Actinomycin D to suppress transcriptional responses during protoplasting has been proposed as a means to reduce these artifacts (33). Alternatively, label-free imaging combined with real-time deep learning–based interpretation of multidimensional cell morphology could be used to assess cell preparation quality prior to collection (34). Protoplasting also results in the loss of spatial context. Integrating single-cell datasets with spatial transcriptomics and metabolomics could help resolve such ambiguities (35).

The relatively low throughput of this method (approximately 200 to 400 cells per experiment) also raises the possibility that rare cell types may be missed. This low throughput is in part because the scMS approach incorporates a chromatographic step in the analytical workflow. However, while this limits the total number of cells that can be analyzed, this approach enabled rigorous identification and quantification of targeted metabolites. Another potential limitation lies in the sensitivity of the mass spectrometry instrumentation. Newer instruments with enhanced sensitivity and faster scan rates could provide deeper coverage of the metabolic landscape. We also note that the limit of detection and quantification of an analyte depends not only on its intrinsic ionization efficiency but also on matrix effects, where compounds in complex mixtures compete for ionization, thereby reducing signal intensity. Therefore, for targeted analyses, sensitivity could be further improved by analyzing cell lysates on a tandem mass spectrometer.

In this work, we quantified specialized metabolites from three chemical classes—iridoids, monoterpene indole alkaloids (MIAs), and flavonoids. While these represent some of the most abundant compounds in *C. roseus* leaves, our untargeted metabolomics dataset revealed more than 800 additional metabolites, demonstrating broad coverage of the chemical space. In our previous study on callus-derived single cells, we showed that scMS enables detection and quantification of triterpenic acids (36), and recent studies further highlight the potential for lipidomics (37, 38) as well as for profiling primary metabolites (30). This suggests that this multiplexing approach can be applied to address a wide range of questions in plant metabolism. Despite the limitations discussed above, we believe this method provides a powerful means to explore correlations between gene expression and metabolite levels and localization at single-cell resolution, adding an important dimension to our understanding of plant metabolism. We anticipate that the datasets generated using this method will facilitate the identification of biosynthetic genes involved in specialized metabolism. Moreover, applying this methodology to other plant systems will enable comparisons of how gene expression and metabolite storage are conserved across different metabolic pathways.

## Materials and Methods

### Plant Growth Conditions.

*C. roseus* plants (Sunstorm Apricot cultivar) were germinated and grown in a York chamber at 23 °C, under a 16 h:8 h, light: dark cycle. Leaves of *C. roseus* were harvested from approx. 2-mo-old plants just before the experiment.

### Chemicals and Reagents.

Commercially available analytical standards used in this study included: loganic acid (Extrasynthese), secologanin (Sigma Aldrich), mauritianin (BIOMOL GmbH), ajmalicine (Sigma Aldrich), catharanthine (Abcam), deacetylvindoline (Toronto Research Chemicals Inc), anhydrovinblastine disulfate (Toronto Research Chemicals Inc.), vinblastine sulfate (Thermo Scientific Chemicals), serpentine hydrogen tartrate (from Sequoia Research Products Ltd.), ajmaline (Extrasynthese), tabersonine (TCI, Tokyo Chemical Industry Co. Ltd.), vindolinine (Advanced ChemBlocks Inc.), vindoline (Acros Organics). Strictosidine and vindorosine were synthesized and isolated in our laboratory, as previously reported (12, 14).

For protoplast extraction, cellulase Onozuka R-10 and macerozyme R-10 were obtained from SERVA, while pectinase, mannitol, KCl, MES, and Bovine serum albumin were purchased from Sigma Aldrich. All solvents used in this study were of UHPLC/MS grade.

### Time-Course Bulk Analysis.

#### Sample preparation.

The leaf material was divided into 2 equal parts. One half was snap-frozen in liquid nitrogen for RNA sequencing as well as for metabolomics analysis. The other half was subjected to protoplasting. Protoplast isolation was carried out as described above. The resulting protoplast suspension was collected at 2 time points: 2.5 and 4 h after incubation of the leaf strips. Aliquots of the cell suspension were snap-frozen upon collection and also used for RNA sequencing and metabolomic analysis.

#### Preparation of samples for metabolomics analysis.

For bulk tissue samples, leaf strips used for protoplast isolation were ground to a fine powder using a TissueLyser II (Qiagen). Metabolites were extracted from 10 mg of the powdered leaf sample with 300 µL of pure MeOH containing 20 nM ajmaline as an internal standard. After vortexing and sonication for 10 min, the leaf extracts were filtered and diluted 500-fold. For bulk protoplast samples, 20 µL of C. roseus leaf protoplast suspension was extracted with 500 µL of pure MeOH containing 20 nM ajmaline as an internal standard. After sonication (10 min) and vortexing, the protoplast extract was filtered, diluted twofold before the analysis.

#### Bulk RNA-sequencing.

mRNA was purified from total RNA using poly-T oligo attached magnetic beads and fragmented. Sequencing libraries were generated from cDNA after end repair, A-tailing, adapter ligation, and size selection. Adapter sequences or poly-A tail from fastq files were trimmed based on fastp, aligned onto genome by STAR (v2.7.10a), and quantified by RSEM (v.1.3.1). Differentially expressed genes were inferred based on edgeR based on the negative binomial distribution model, and genes that showed |log2(FoldChange)| ≥ 1 & padj ≤ 0.05 were regarded as differentially expressed (Dataset S01) (39). ClusterProfiler was used for GO enrichment analysis (40).

### Single-Cell Collection.

#### Protoplast isolation.

Young leaves (2 to 3 cm) of *C. roseus* plant were rinsed with distilled water, cut in strips with a surgical blade and immediately submerged in digestion medium (2% (w/v) Cellulase Onozuka R-10, 0.3% (w/v) macerozyme R-10, 0.1% (w/v) pectinase, and 0.1% (w/v) bovine serum albumin dissolved in Mannitol-MES (MM) buffer). MM buffer contained 0.45 M mannitol and 20 mM MES, pH 5.7 to 5.8. Digestion medium was infiltrated into the leaf strips by applying vacuum for 15 min (200 mBar), and protoplasts were released from the leaf strips while shaking for 2.5 h at room temperature. The protoplast suspension was strained through 40 μm sieves to remove large debris and transferred to 15 mL round-bottom tubes. Protoplasts were centrifuged at 70×*g* for 5 min and washed with corresponding MM buffer three times.

#### Single-cell picking.

After washing, the protoplasts were resuspended in a small volume of MM buffer. After FDA staining, their concentration and cell viability were checked under a microscope. Single-cell picking was performed as described previously (12). Briefly, approximately 10,000 protoplasts were dispensed onto a Sievewell™ (Sartorius) with cell-size micropores (50 μm) to capture single cells by gentle suction-induced sedimentation. The CellCelector™ Flex (Sartorius) cell picking robot was used to transfer the targeted cells from the Sievewell™ to skirted twin.tec PCR Plates 96 (Eppendorf) where each well contains 8 µL of RNase-free water containing 0.5 U of protector RNase inhibitor (Roche). After being deposited into the destination wells, the cells were lysed by osmotic shock. The resulting cell lysate was divided into duplicates, one for scMS and the other for scRNA-seq, both kept in –80 °C for short-term storage.

### scMS.

#### Metabolomics profiling of single cells using UPLC-MS.

UPLC-MS analysis was performed as described previously on a Vanquish (Thermo Fisher Scientific) system coupled to a Q-Exactive Plus Orbitrap (Thermo Fisher Scientific) mass spectrometer (12). Briefly, chromatographic separation was carried out on a Waters™ ACQUITY UPLC BEH C18 130 Å column (1.7 μm, 1 mm × 50 mm) maintained at 40 °C. The binary mobile phases were 0.1% HCOOH (formic acid) in MilliQ water (aqueous phase) (A) and acetonitrile (ACN) (B). The gradient started with 1% B for 0.5 min, increased linearly to 70% B over 5 min, followed by a wash stage was performed at 99% B for 0.5 min before switching back to 1% B for 1.5 min to condition the column for the next injection. Total chromatographic run time was 7 min with a flow rate of 0.3 mL min^−1^. The injection volume was 4 μL, and the autosampler was kept at 10 °C throughout the analysis.

The Q-Exactive Plus Orbitrap mass spectrometer (Thermo Fisher Scientific) was equipped with a heated electrospray ionization (HESI) source. Acquisition was performed in full scan MS mode (resolution 70,000-FWHM at 200 Da) in positive mode over the mass range *m/z* from 120 to 1,000. The full-scan and data-dependent MS/MS mode (full MS/dd-MS^2^ Top10) was used for QC pooled samples to simultaneously record the spectra of the precursors as well as their MS/MS (fragmentation) (*SI Appendix*, Fig. S21). In addition, the full MS/dd-MS^2^ mode with inclusion list was also applied for the pooled QC samples to confirm fragments of the selected precursors. The parameters for dd-MS^2^ were set up as follows: resolution 17,500, mass isolation window 0.7 Da, and normalized collision energy (NCE) was set at 3 levels: 15, 30, and 45%. Spectrum data format was centroid and all the parameters of the UHPLC-HRMS system were controlled through Xcalibur software version 4.3.73.11 (Thermo Fisher Scientific).

#### LC–MS data processing and analysis.

For targeted analysis, peak area from extracted ion chromatograms (EIC) was integrated and extracted using the Xcalibur Quan Browser version 4.3.73.11 (Thermo Fisher Scientific). For untargeted analysis, raw data were imported into Compound Discoverer™ software 3.2 (Thermo Fisher Scientific) for peak picking, deconvolution, and formula assignment. Parameters are listed in *SI Appendix*, Table S5.

#### Quantification for targeted compounds in single cells.

The identification of targeted analytes in the samples was confirmed by comparing retention times to authentic reference standards. Standard solutions were prepared in MeOH at an approximate concentration of 1 mM, with the exact concentration recorded. These solutions were serially diluted down to 0.001 nM and analyzed by UHPLC-MS to determine limit of quantification (LOQ) and calibration range (*SI Appendix*, Table S1). Each calibration point was measured in triplicate, and linear regression curves were calculated using peak areas.

Quantification of analytes in the scMS cell lysate was performed using external calibration curves. Since the lysate was split in half, one for scMS and one for scRNA-seq, the analyte amount measured in one portion (for scMS) was doubled to calculate the total amount in a single protoplast. The analyte concentrations in each protoplast were then calculated by dividing the absolute amounts of analytes detected by the estimated volume of the cell, which was calculated assuming the protoplasts are spherical. Protoplast diameters were measured using ImageJ software, based on images obtained from the cell picking robot. Empty wells, in which no cell was present, were analyzed to determine the background levels of the metabolites.

### Single-Cell RNA Sequencing.

#### Sequencing library construction and read alignment.

Single-cell mRNA sequencing libraries were prepared with a half of cell lysate using the SMART-Seq mRNA LP (Takara, 634771) and the customized automation program on Biomek i7 hybrid liquid handler (Beckman Coulter, B87585) according to standard manufacturer’s protocol (PCR 1: 18 cycles and PCR 2: 16 cycles, individually purified). The final library quantification and quality check were performed using the Qubit Flex Fluorometer (Thermo Fisher Scientific, Q33327), 4200 TapeStation System (Agilent, G2991BA), and LightCycler 480 II (Roche, 05015278001). The libraries were sequenced on Illumina NovaSeq 6000 (2 x 60 bp) and demultiplexed by bcl2fastq Conversion Software (Illumina) to obtain FASTQ data files.

Index sequences for each fastq file were verified whether they match their corresponding 96-well positions. After confirming qualities of sequencing files via fastqc, adaptors, poly-A tails, and low-quality bases were trimmed out of sequencing reads using fastp (41). The cleaned reads were aligned to reference genome (cro_v3) via STAR (v.2.7.10a) and quantified using RSEM (v.1.3.1) (42, 43). The output expected counts were combined into one matrix and subjected to Seurat (v5.0.1) for downstream analyses (44).

#### Processing of single-cell transcriptome.

Empty wells or duplets were excluded, and cells containing more than 1,000 genes and less than 10,000 genes were kept for downstream analyses. Read counts were log-normalized, and 500 variable genes were selected by the vst method and scaled for transcriptome-guided dimensional reduction. Genes previously validated by RNA in situ hybridization worked as markers for cell type annotation (*NLTP2*, *CB21*, *D4H*, *ISY*, and *G8H*). Cells that have detectable *G8H* or *ISY* were assigned as IPAP cells, and cells that express *D4H* or *DAT* were regarded as idioblasts.

### Comparing Transcriptome and Metabolome.

#### Integration.

Before integrating the transcriptomic and metabolomic datasets, empty wells or wells containing doublet of cells were removed based on the images recorded by CellCelector. The estimated concentrations of the targeted metabolites or peak areas of untargeted metabolites were log_10_-normalized and standardized within the batch before appended to the Seurat transcriptome object as additional assays. Log-normalized matrices as data and z-scored ones served as scale.data. The presence of marker molecules (secologanin, loganic acid, and serpentine) was used as a marker for metabolite-guided cell type annotation as previously described (12). Concentrations of twelve compounds were used as features for metabolite-guided dimensional reduction, and localization of compounds was used for annotating each cluster in metabolite-guided UMAP. Feature names of untargeted metabolites were given by concatenating their formula and retention time detected. Well identity of each cell was utilized to connect their corresponding UMAP coordinates from transcriptome and metabolome-guided cell clustering.

#### Gene by metabolite correlation.

Compound concentration and TPM of genes in each cell were used to calculate Spearman correlation between genes and metabolites (Dataset S02). Calculated coefficients of all genes with each specific compound were visualized in the scatter plot according to their ascending rank or in heatmap where the coefficients were shown in color scale. Information regarding previously reported genes is listed in Dataset S03. Transporters with the top 15 highest and bottom 15 lowest correlation values were connected to corresponding compounds and visualized in the network using Cytoscape (45).

#### Comparison of differentially expressed genes between subclusters.

Cells accumulating secologanin alone and cells accumulating both secologanin and mauritianin were annotated as each subcluster in the epidermis. Differentially expressed genes between two subclusters were extracted using a Wilcoxon rank-sum test.

## Supplementary Material

Appendix 01 (PDF)

Dataset S01 (XLSX)

Dataset S02 (XLSX)

Dataset S03 (XLSX)

## Data Availability

All data used in this study are available in the article and/or supporting information. Sequencing data have been deposited in NCBI (PRJNA1248169) (46). Custom code developed for this work is available in the GitHub repository (https://github.com/moonyoungkang/split_cell_2025) (47). The Cytoscape file containing the raw data for Fig. 4 is available at https://doi.org/10.5281/zenodo.17091477 (48).
