# Supplementary material for: Single-cell metabolome and RNA-seq multiplexing on single plant cells
Source: Proc Natl Acad Sci U S A. 2025 Oct 24;122(43):e2512828122. doi: 10.1073/pnas.2512828122 (PMC12582292; doi:10.1073/pnas.2512828122)
Supplement: Supplementary file 1 — Appendix 01 (PDF) [file pnas.2512828122.sapp.pdf]

Supporting Information for

Single-cell metabolome and RNA-seq multiplexing on single plant cells

Moonyoung Kang[†], Anh Hai Vu[†], Abbie L. Casper, Rinho Kim, Jens Wurlitzer, Sarah Heinicke, Assa Yeroslaviz, Lorenzo Caputi*, and Sarah E. O'Connor*

[†] These authors contributed equally to this work

Corresponding authors: Lorenzo Caputi and Sarah E. O'Connor

Emails: lcaputi@ice.mpg.de or oconnor@ice.mpg.de

This PDF file includes:

Supplementary Scheme 1

Figures S1 to S21

Tables S1 to S5

SI References

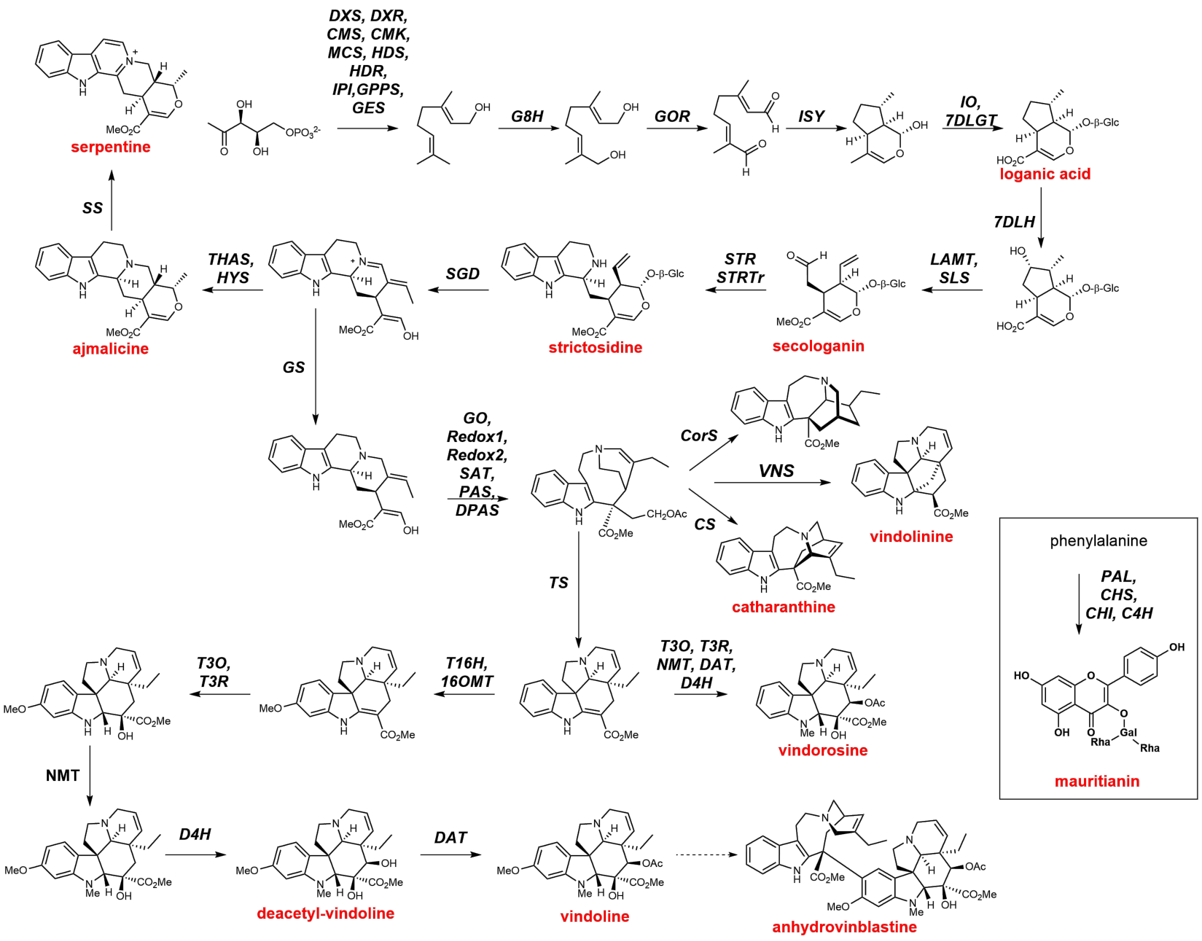

Supplementary Scheme 1. A complete biosynthetic pathway to metabolites of *Catharanthus roseus*.

Key metabolites targeted in this study are highlighted in red text. All related biosynthetic genes for these steps are indicated in black, with solid lines. Unconfirmed step is shown as dashed line. The pathway to mauritianin, which is unrelated to other metabolites, is shown inside inset. Gene abbreviations: *DXS*, 1-deoxy-D-xylulose 5-phosphate synthase; *DXR*, 1-deoxy-D-xylulose-5-phosphate reductoisomerase; *CMS*, 4-diphosphocytidyl-methylerythritol 2-phosphate synthase; *CMK*, 4-diphosphocytidyl-2-C-methyl-D-erythritol kinase; *MCS*, 2C-methyl-D-erythritol 2,4-cyclodiphosphate synthase; *HDS*, 1-hydroxy-2-methyl-2-(E)-butenyl-4-diphosphate synthase; *HDR*, 1-hydroxy-2-methyl-butenyl 4-diphosphate reductase; *IPI*, isopentenyl pyrophosphate:dimethylallyl pyrophosphate isomerase; *GPPS*, geranyl pyrophosphate synthase; *GES*, geraniol synthase; *G8H*, geraniol 8-hydroxylase; *GOR*, 8-hydroxygeraniol oxidoreductase; *ISY*, iridoid synthase; *IO*, iridoid oxidase; *7DLGT*, UDP-glucose iridoid glucosyltransferase; *7DLH*, 7-deoxyloganic acid 7-hydroxylase; *LAMT*, loganic acid methyltransferase; *SLS*, secologanin synthase; *STR*, strictosidine synthase; *STRTr*, strictosidine transporter; *SGD*, strictosidine b-glucosidase; *THAS*, tetrahydroalstonine synthase; *HYS*, heteroyohimbine synthase; *SS*, serpentine synthase; *GS*, geissoschizine synthase; *GO*, geissoschizine oxidase; *Redox1*, reductive enzyme 1; *Redox2*, reductive enzyme 2; *SAT*, stemmadenine acetyl transferase; *PAS*, precondylocarine acetate synthase; *DPAS*, dihydroprecondylocarpine synthase; *CorS*, coronaridine synthase; *VNS*, vindolinine synthase; *CS*, catharanthine synthase; *TS*, tabersonine synthase; *T16H*, tabersonine 16-hydroxylase; *16OMT*, 16-hydroxytabersonine O-methyltransferase; *T3O*, tabersonine 3-oxidase; *T3R*, tabersonine 3-reductase; *NMT*, 16-hydroxy-2,3-dihydro-3-hydroxytabersonine N-methyltransferase; *D4H*, desacetoxyvindoline-4-hydroxylase; *DAT*, deacetylvindoline 4-O-acetyltransferase; *PAL*, phenylalanine ammonia lyase; *CHS*, chalcone synthase; *CHI*, chalcone isomerase; *C4H*, cinnamate-4-hydroxylase.

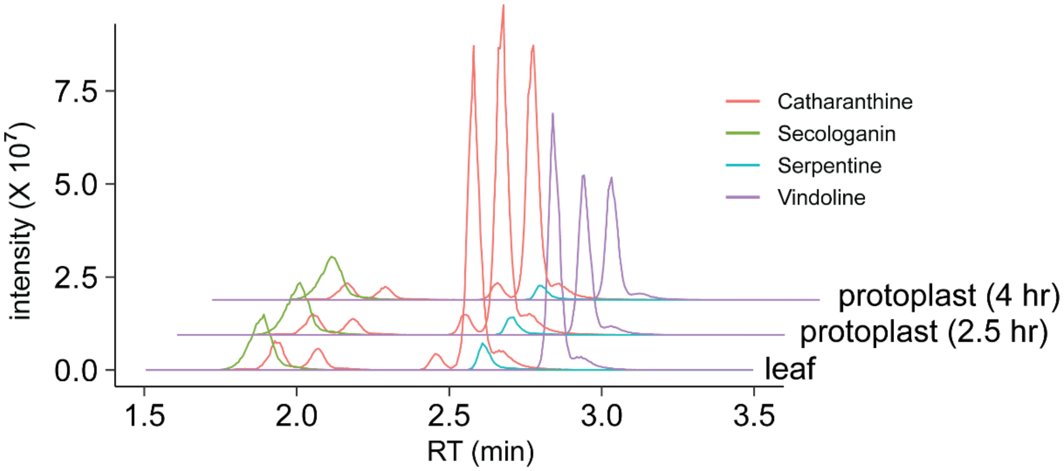

Fig. S1: Comparison of four alkaloids in the leaf tissue extract and in the protoplasts extract.

Extracted ion chromatograms for the compounds catharanthine, secologanin, serpentine, vindoline in leaf tissues used for producing protoplasts and the protoplast suspension after 2.5 h to 4 h post-incubation.

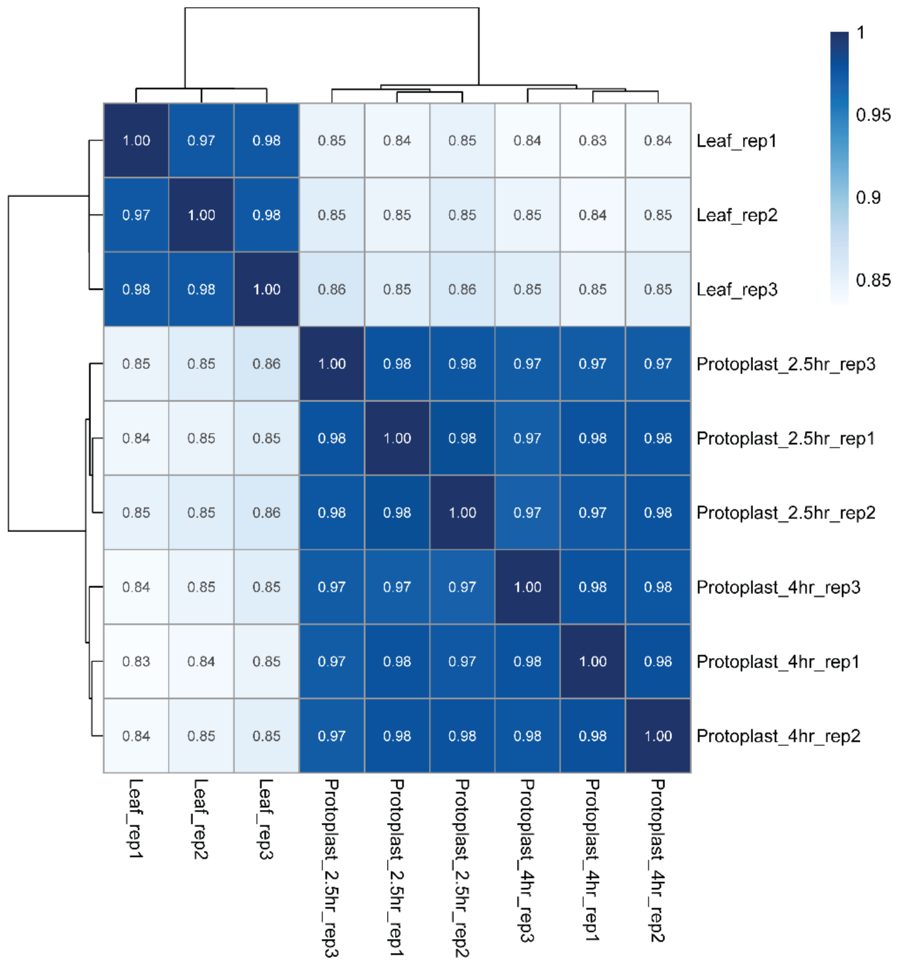

Fig. S2: Spearman correlation of bulk transcriptome.

Bulk transcriptome of *C. roseus* leaf before protoplasting, and protoplasts after 2.5 h and 4 h with additional 90 minutes incubation were compared based on Spearman correlation. Protoplasts were stable after 90 minutes-incubation in protoplast wash solution.

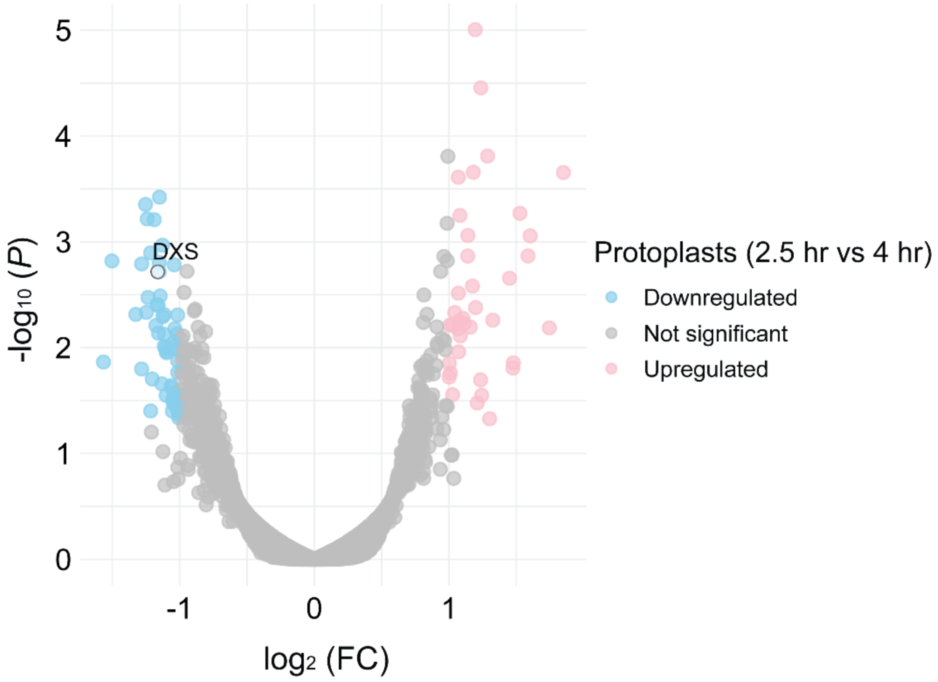

Fig. S3: Volcano plot of protoplasts (2.5 h and 4 h).

Volcano plot was plotted according to edgeR result, where differentially expressed genes (|log2(FoldChange)| ≥ 1 & padj ≤ 0.05) were plotted with corresponding colors. Biosynthetic genes or transcription factors characterized in previous studies are outlined in black.

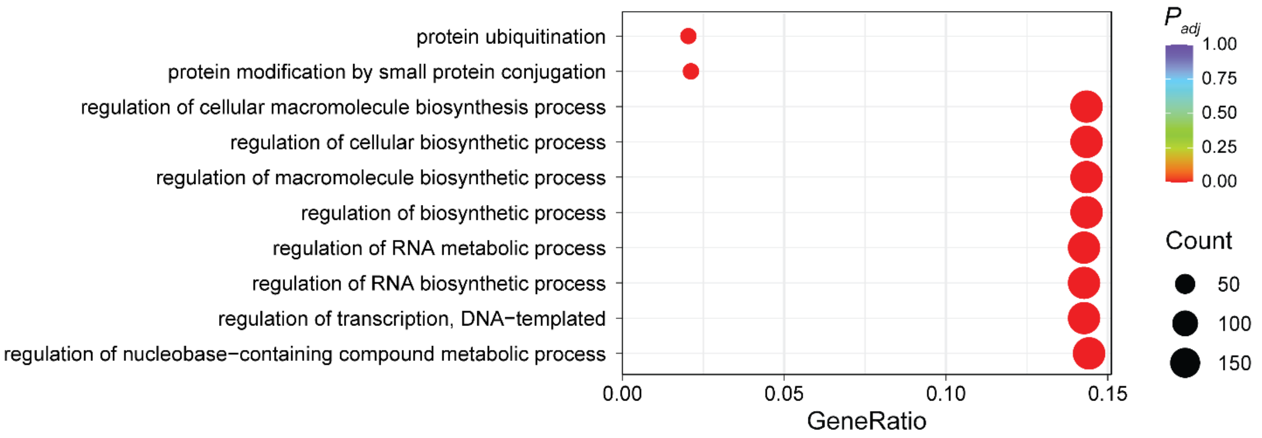

Fig. S4: GO term enrichment (Biological process) of differentially expressed genes.

Top 10 significant GO terms of upregulated genes in protoplasts (2.5 h) are shown with number of genes (size) and P_{adj}. Gene ratio refers to the ratio of differentially expressed genes to total genes that have each GO.

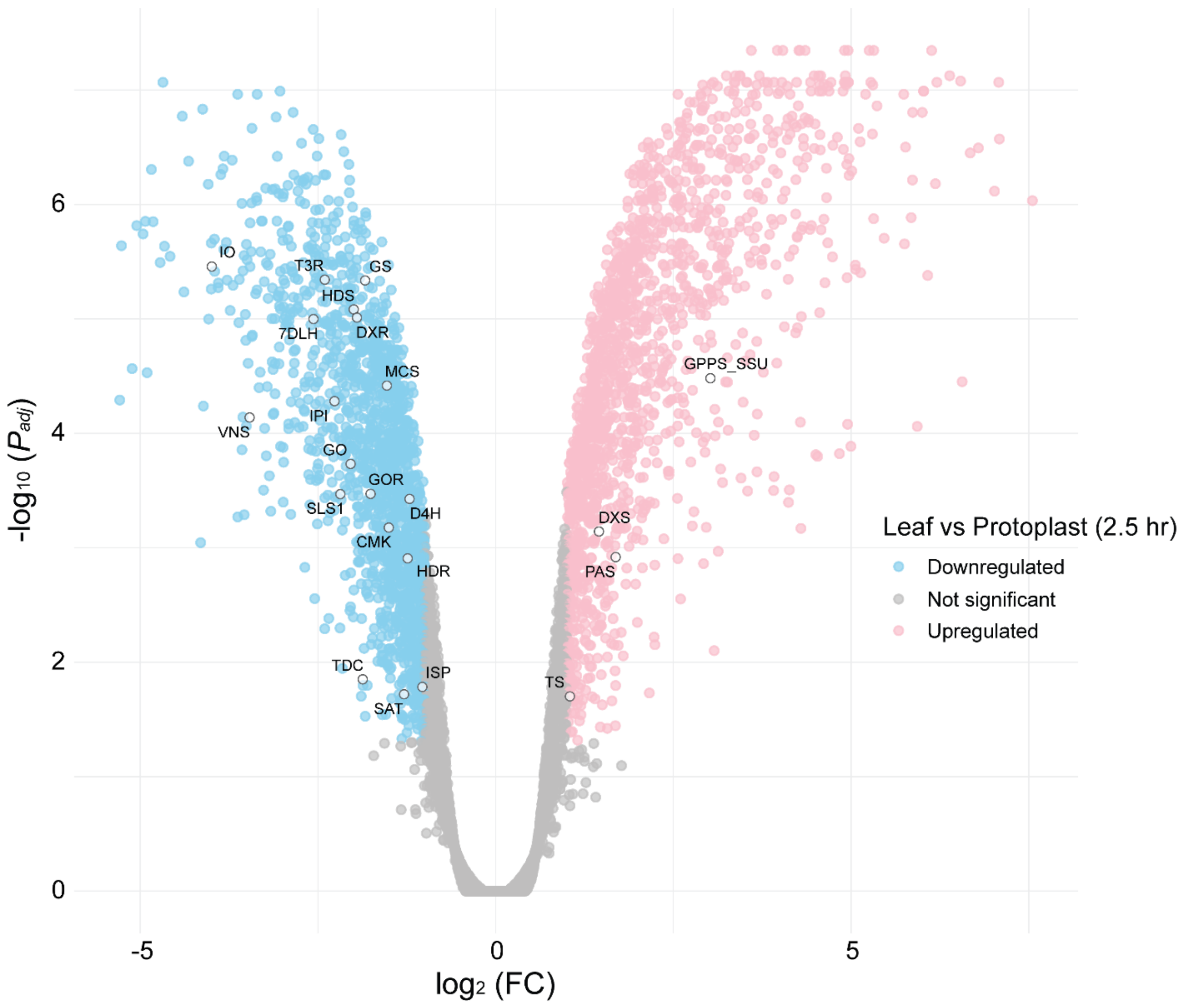

Fig. S5: Volcano plot of leaf and protoplasts (2.5 h).

Volcano plot was plotted according to edgeR result, where differentially expressed genes (|log2(FoldChange)| ≥ 1 & padj ≤ 0.05) were plotted with corresponding colors. Biosynthetic genes characterized in previous studies are outlined in black.

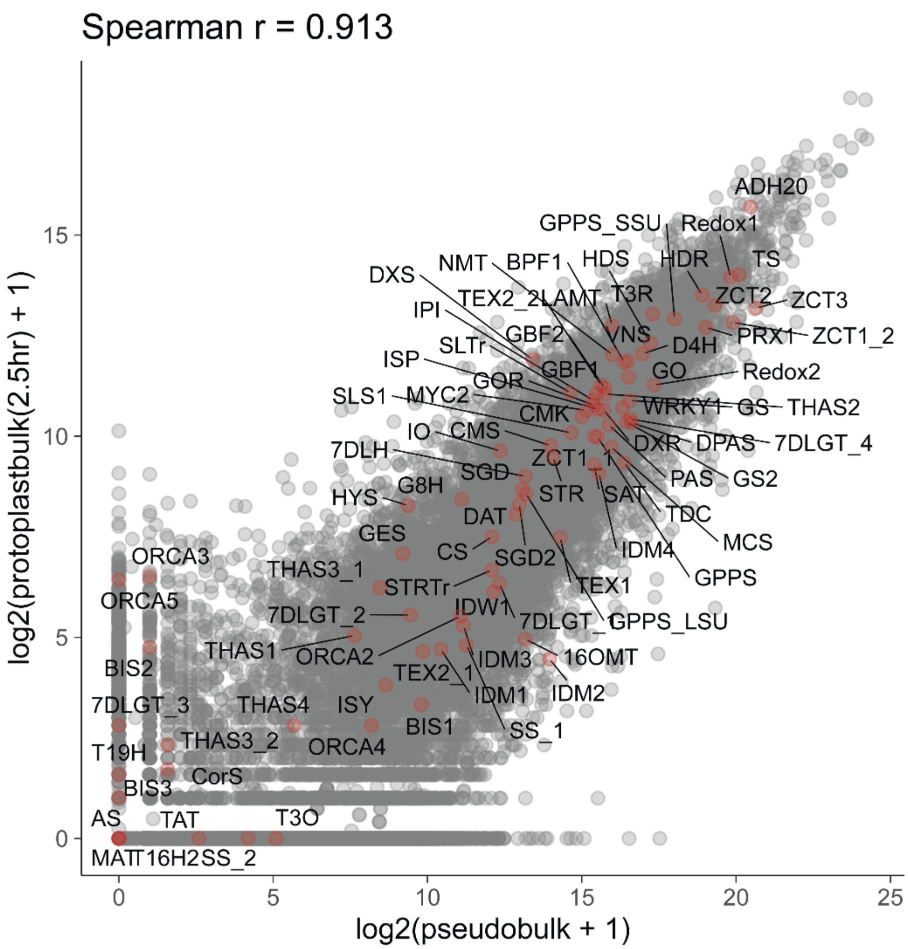

Fig. S6: Scatter plot of protoplast (bulk 2.5 h) and pseudobulk of single cell transcriptome.

Pseudobulk was generated by aggregating counts of single cells, and compared with bulk transcriptome. Biosynthetic genes or transcription factors characterized in previous studies are colored red. Spearman correlation coefficient = 0.913.

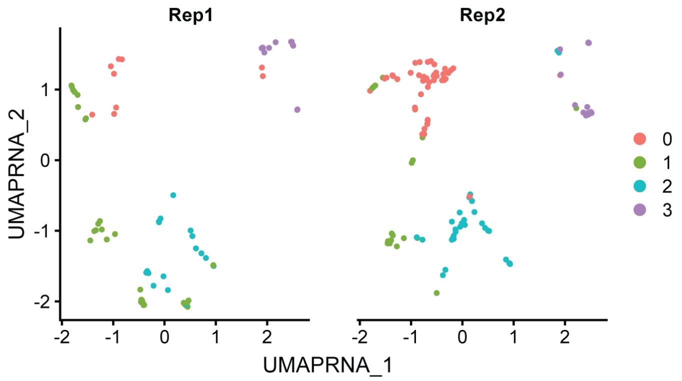

Fig. S7. Datasets for single cell RNA sequencing replicates.

After processing single cell RNA sequencing data of cells, their locations were split by original replicate information. The two batches are integrated on UMAP without any significant bias.

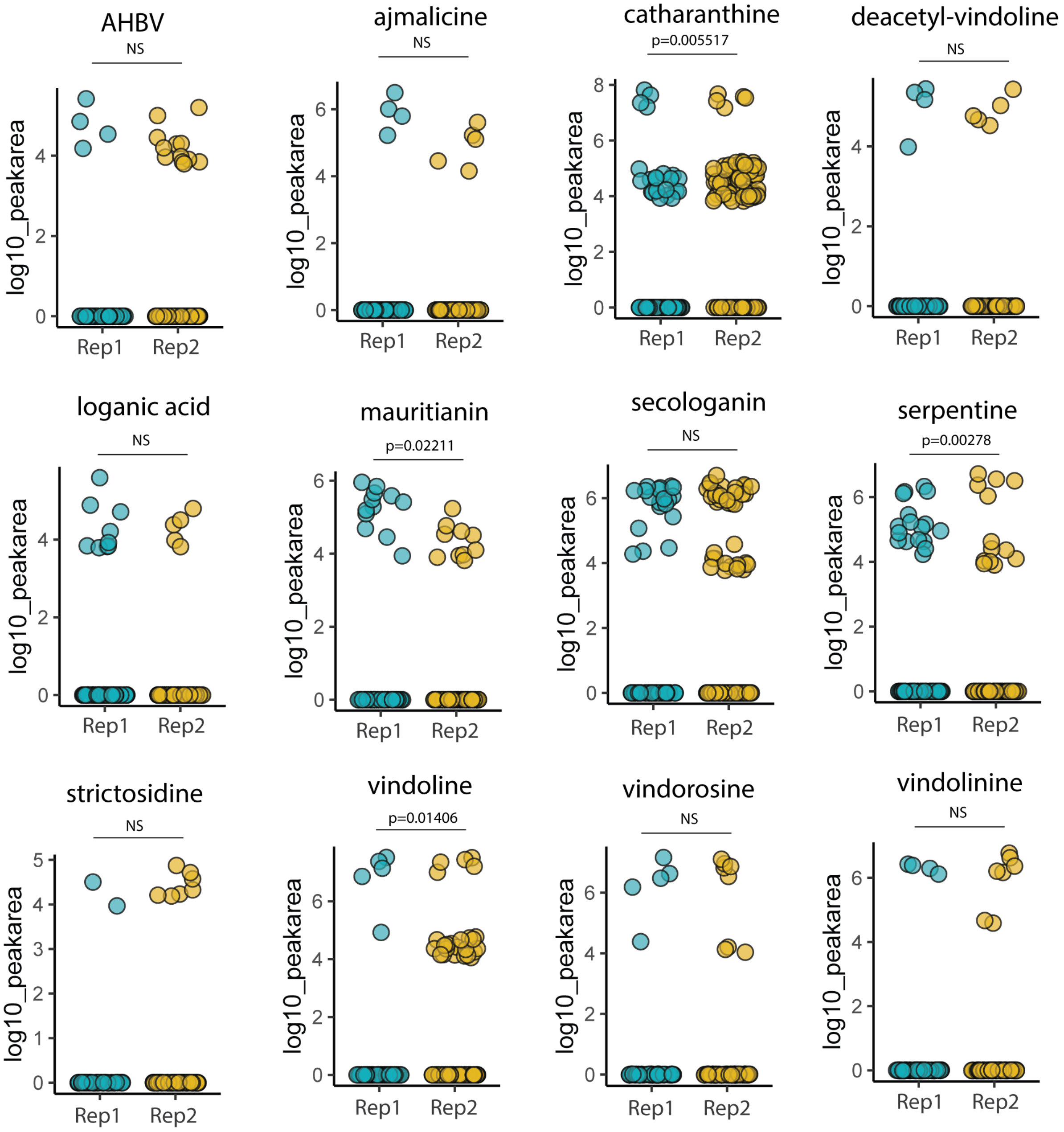

Fig. S8. Comparison of the peak areas of targeted analytes from two replicates.

Detected peak areas of targeted analytes were compared between two biological replicates. Most of the analytes did not show a statistical difference between replicates. Numbers of cells that contained vindoline, serpentine, mauritianin, and catharanthine showed differences between replicates, but we assume that this is due to the relatively small number of cells sampled (74 cells in rep1, 119 cells in rep2). Statistical differences between the two replicates were calculated by performing two-sided KS tests (*P* values are annotated; NS, not significant).

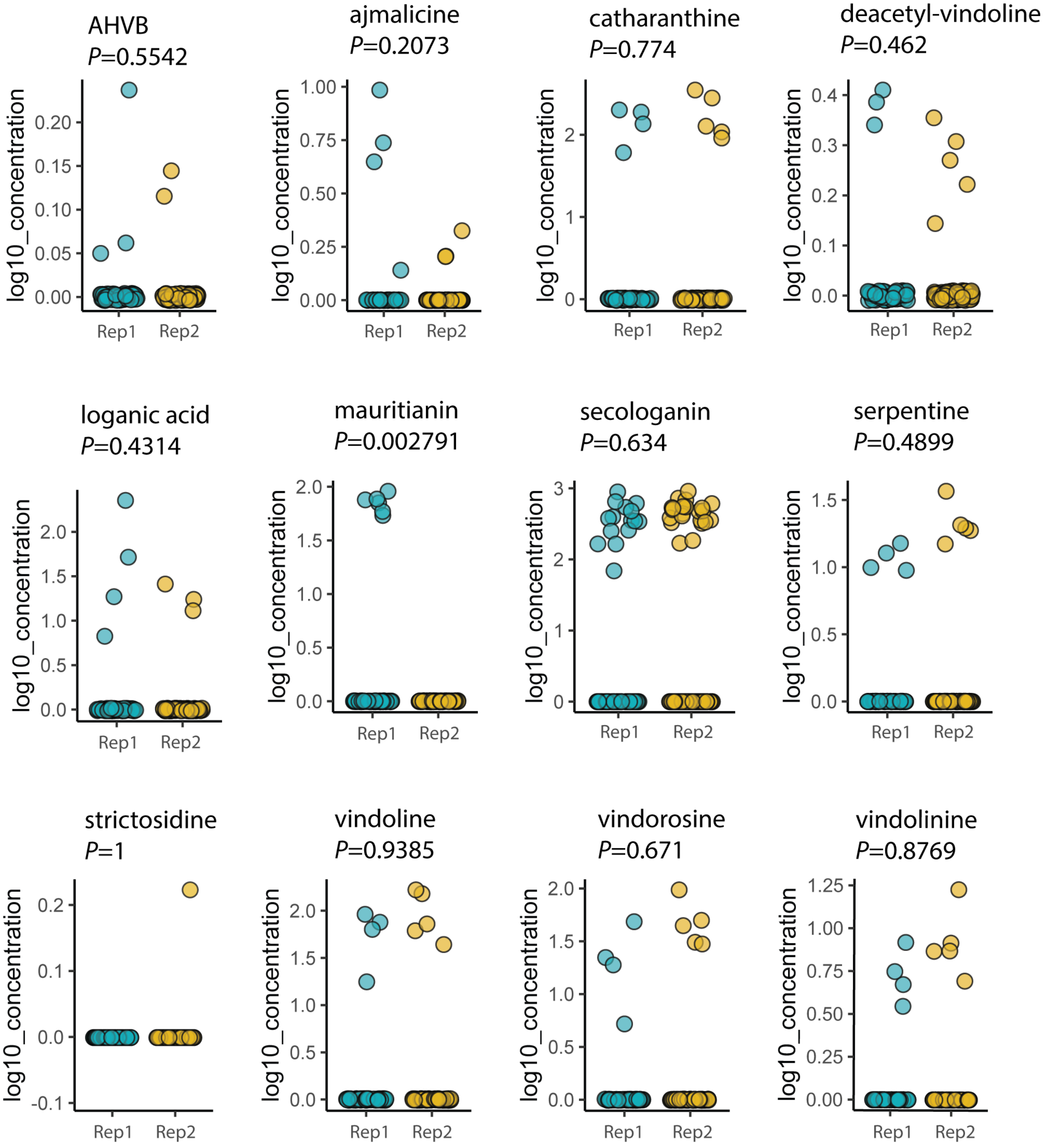

Fig. S9. Comparison of the absolute concentration of targeted analytes from two replicates (Concentration).

Calculated intra-cellular concentrations of targeted analytes were compared between two biological replicates. Most of the analytes did not show a statistical difference between replicates. Numbers of cells that contained vindoline, serpentine, mauritianin, and catharanthine showed differences between replicates, but we assume that this is due to the relatively small number of cells sampled (74 cells in rep1, 119 cells in rep2). Statistical differences between the two replicates were calculated by performing two-sided KS tests (*P* values are annotated).

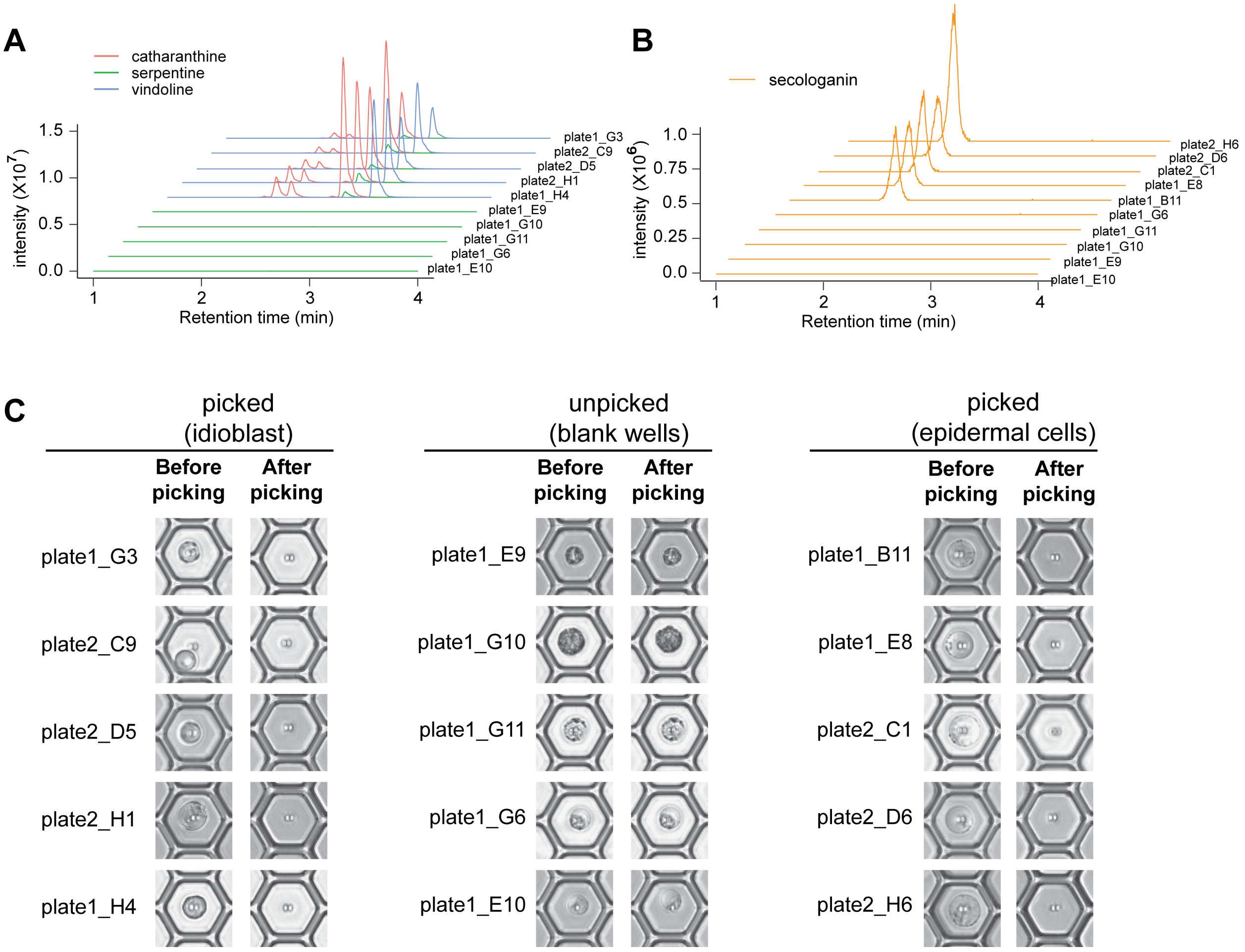

Fig. S10. Representative chromatograms of idioblast and epidermal cells

A. Alkaloids (catharanthine, serpentine, vindoline) detected in idioblast cells are shown as extracted ion chromatograms with blank wells where cells were not picked. B. Secologanin detected in epidermal cells. C. Cells picked by CellCelector. Photos were taken before picking and after picking, enabling the observation of cellular morphology and the assessment of whether cells had been successfully collected or not.

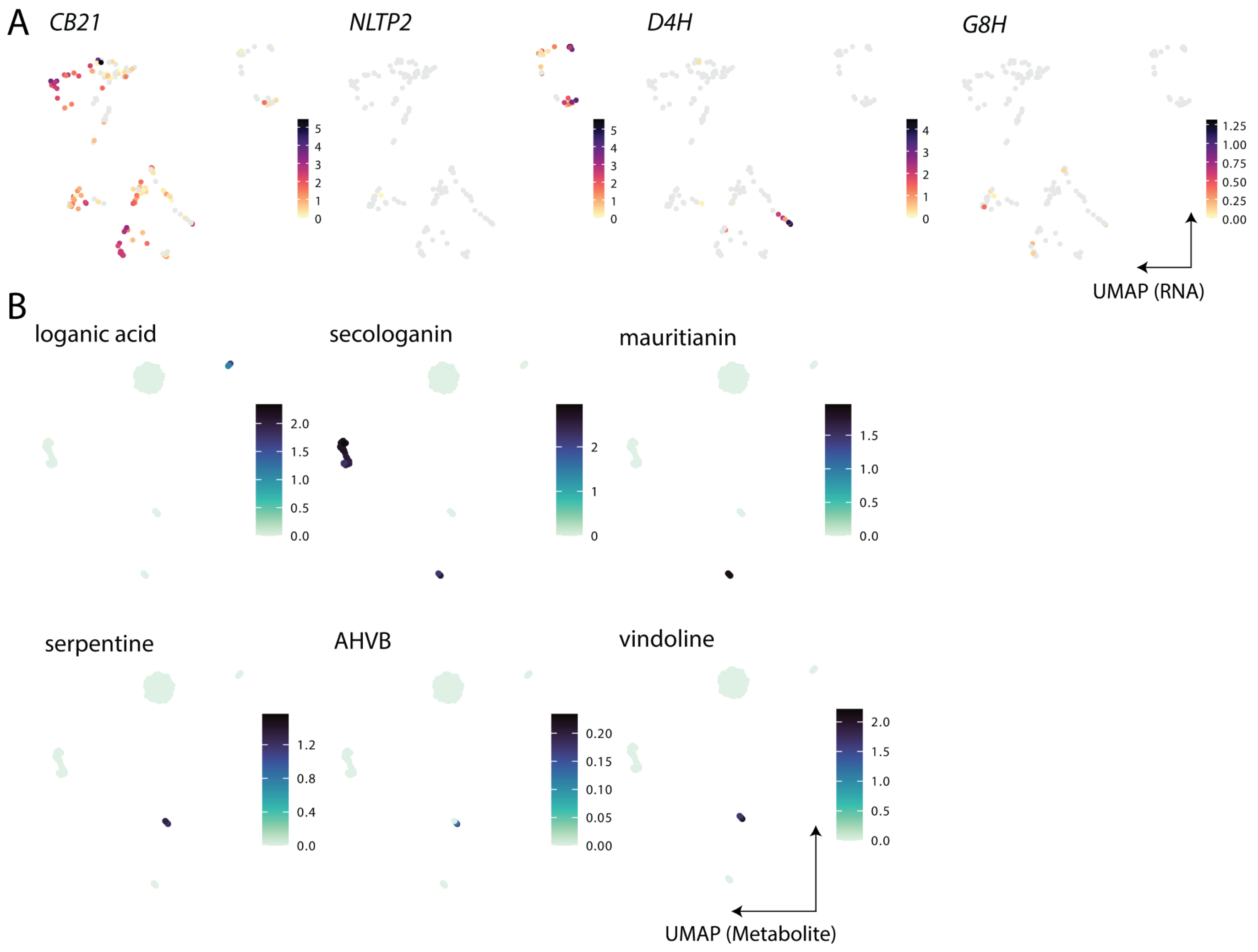

Fig. S11. Representative marker genes and analytes that are used for annotating two UMAP plots.

A. *CB21* (parenchyma), *NLTP2* (epidermis), *D4H* (idioblast), and *G8H* (IPAP) expressions on RNA UMAP. **B.** loganic acid (IPAP), secologanin and mauritianin (epidermis), serpentine, vindoline, and AHVB (idioblast) accumulation on MET-UMAP.

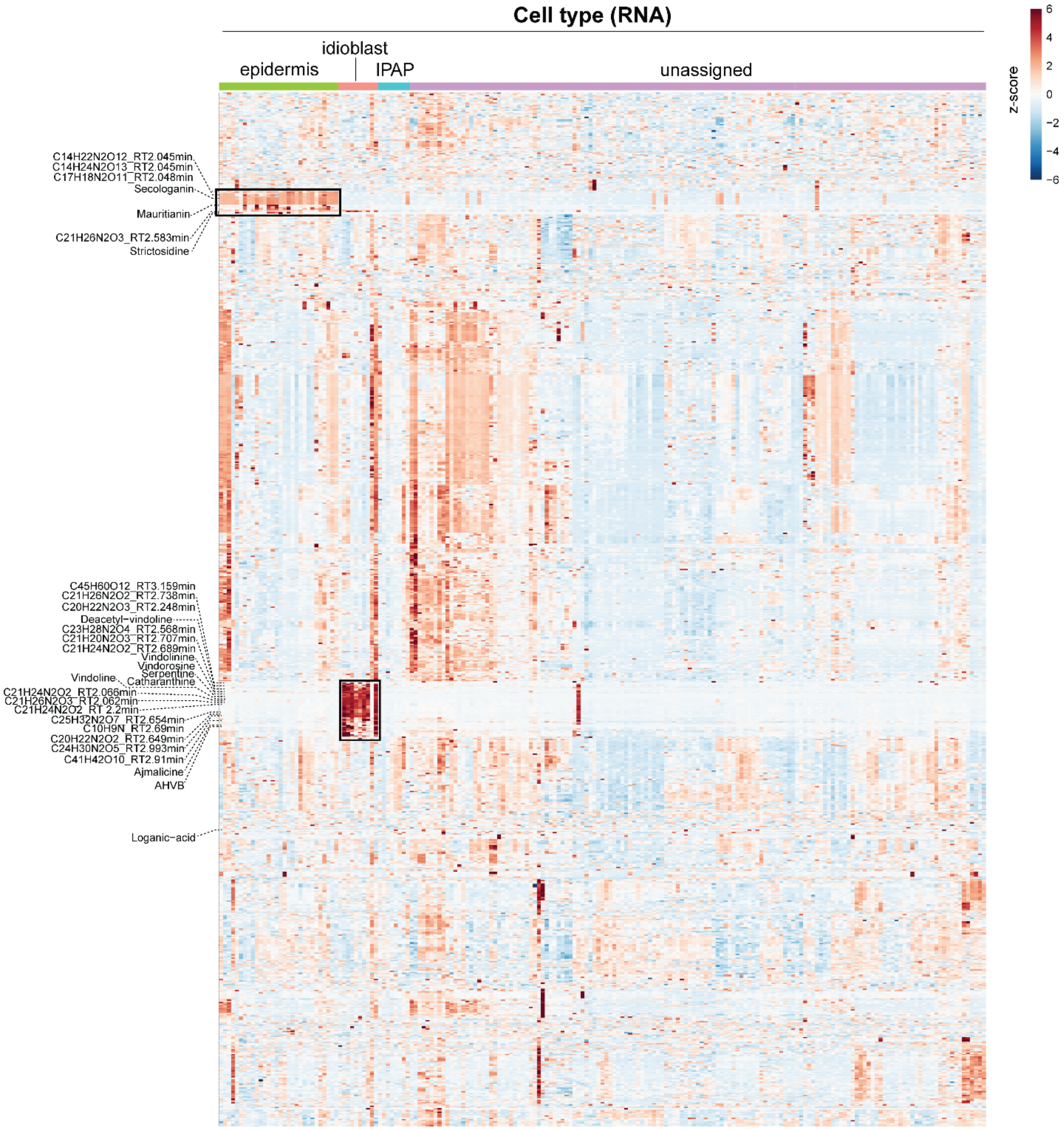

Fig. S12: Metabolites used in cell clustering.

All metabolites (targeted and untargeted) used in cell clustering are plotted as a heatmap based on z-score. Column represents single cell and rows represents analytes. Epidermis and idioblast modules are outlined in black squares with some exemplar names of features.

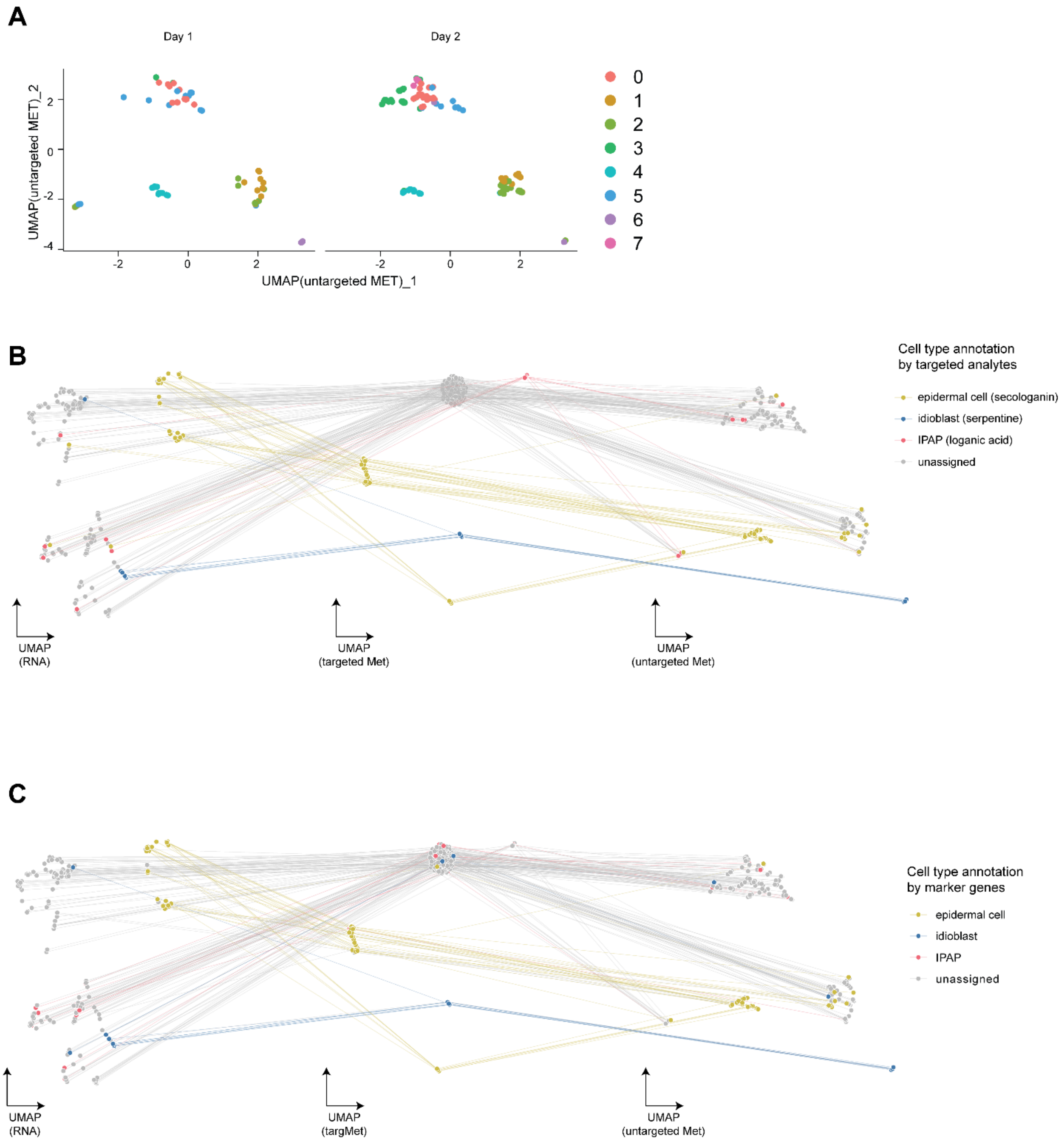

Fig. S13: Cell clustering based on untargeted metabolomic data.

A. Datasets for single cell untargeted metabolomic data, two replicates. The two batches are integrated on UMAP without any significant bias. B, C. Three different UMAP plots were connected based on their corresponding well identities, where cells are colored with annotations based on targeted metabolite accumulation (serpentine, secologanin, mauritianin, loganic acid), or marker gene expressions.

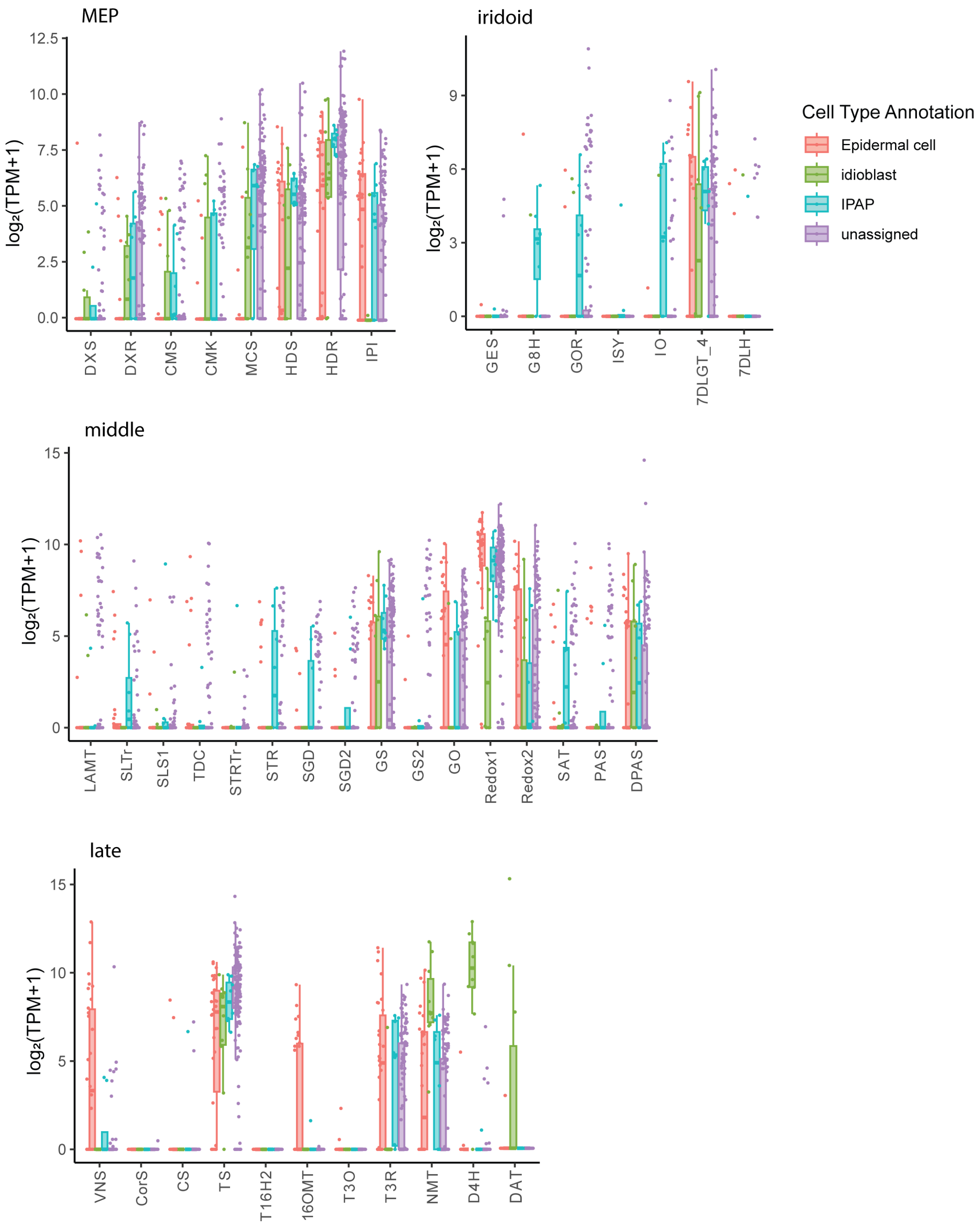

Fig. S14. Boxplot showing expression of MIA biosynthetic genes.

Log2-normalized gene expression level is shown as box plot for each gene, grouped and colored by marker gene-guided annotation. The box shows the interquartile range (IQR), and the whiskers correspond to the highest and lowest points within 1.5× IQR.

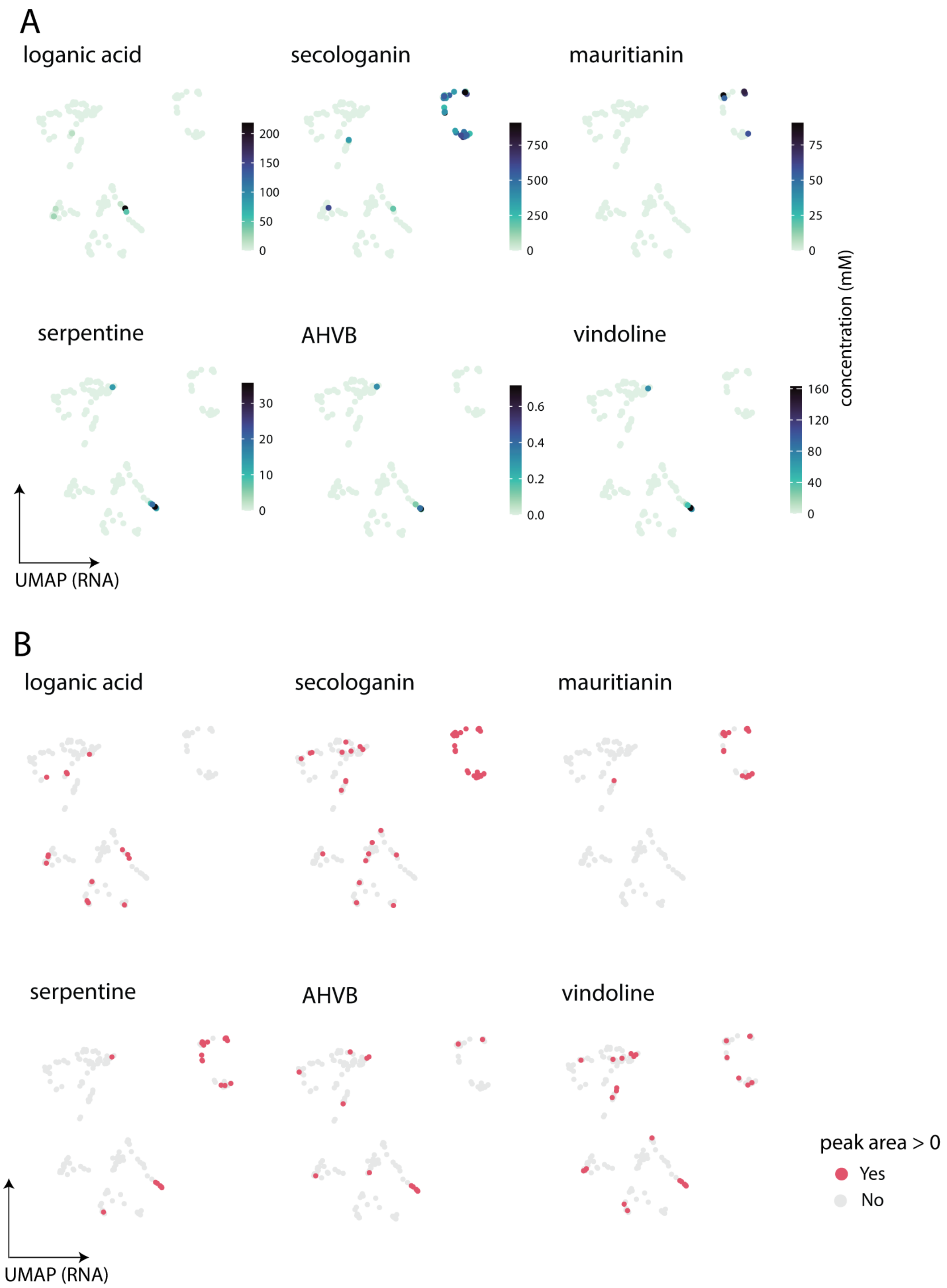

Fig. S15. Location of targeted analyte accumulation on RNA-UMAP.

A. Key analytes are plotted on the RNA-UMAP, showing cell-type specific accumulations. The concentration of each analyte is shown based on color-scale. **B.** Detected peak area are plotted on RNA-UMAP. Secologanin and serpentine show some leaky pattern of accumulation, outside epidermis and idioblast cells, respectively.

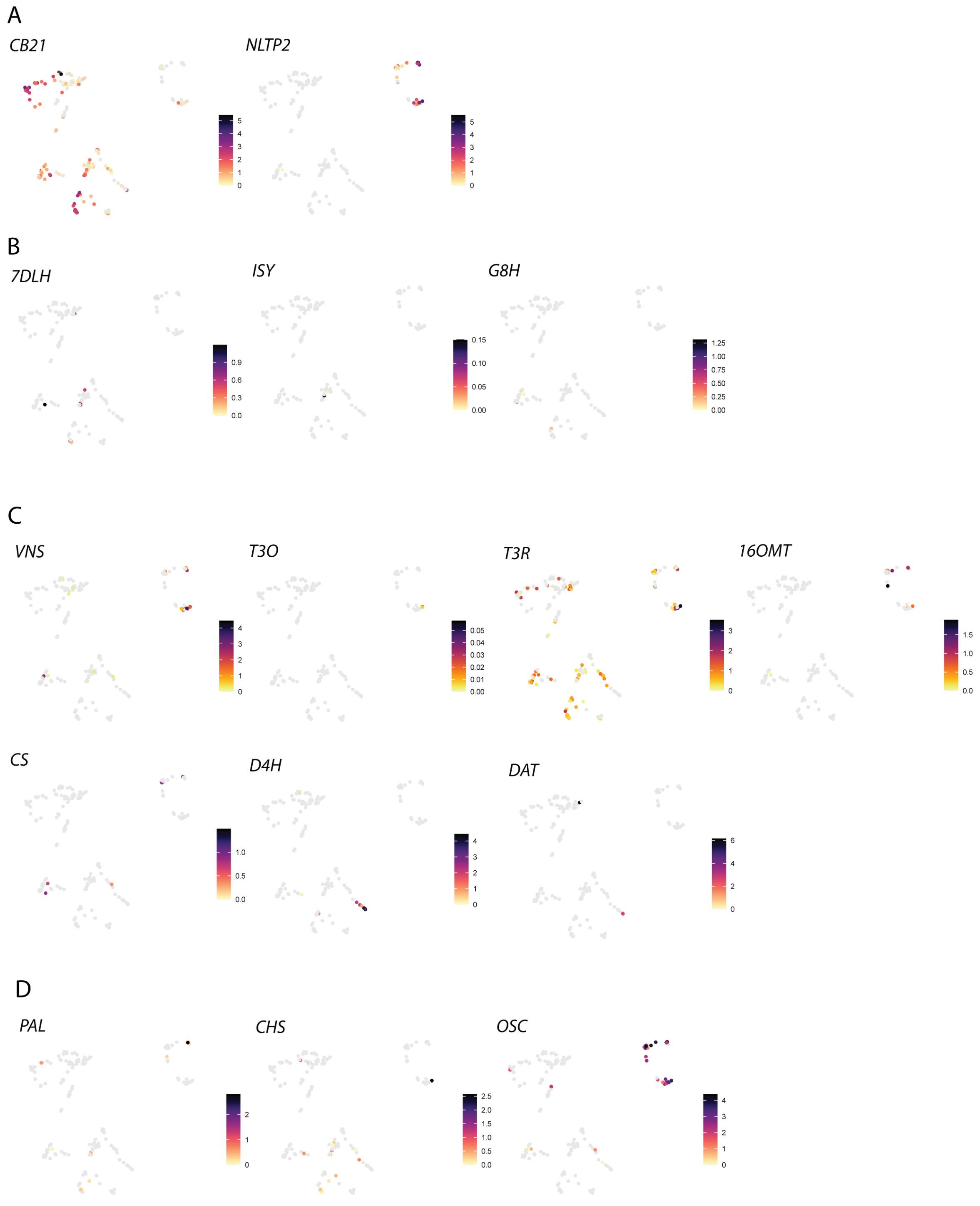

Fig. S16. Feature plots of representative genes on RNA-UMAP.

A. Parenchyma (*CB21*), epidermis marker (*NLTP2*) genes show exclusive expression pattern in each cell type. **B.** Iridoid biosynthetic show scattered pattern without clear coexistence or clustering. **C.** Late alkaloid biosynthetic genes are mostly localized outside idioblasts, except for *D4H* and *DAT*. **D.** Representative phenylpropanoid genes are localized outside epidermis. *OSC* (Oxidosqualene cyclase) is specifically expressed in epidermis.

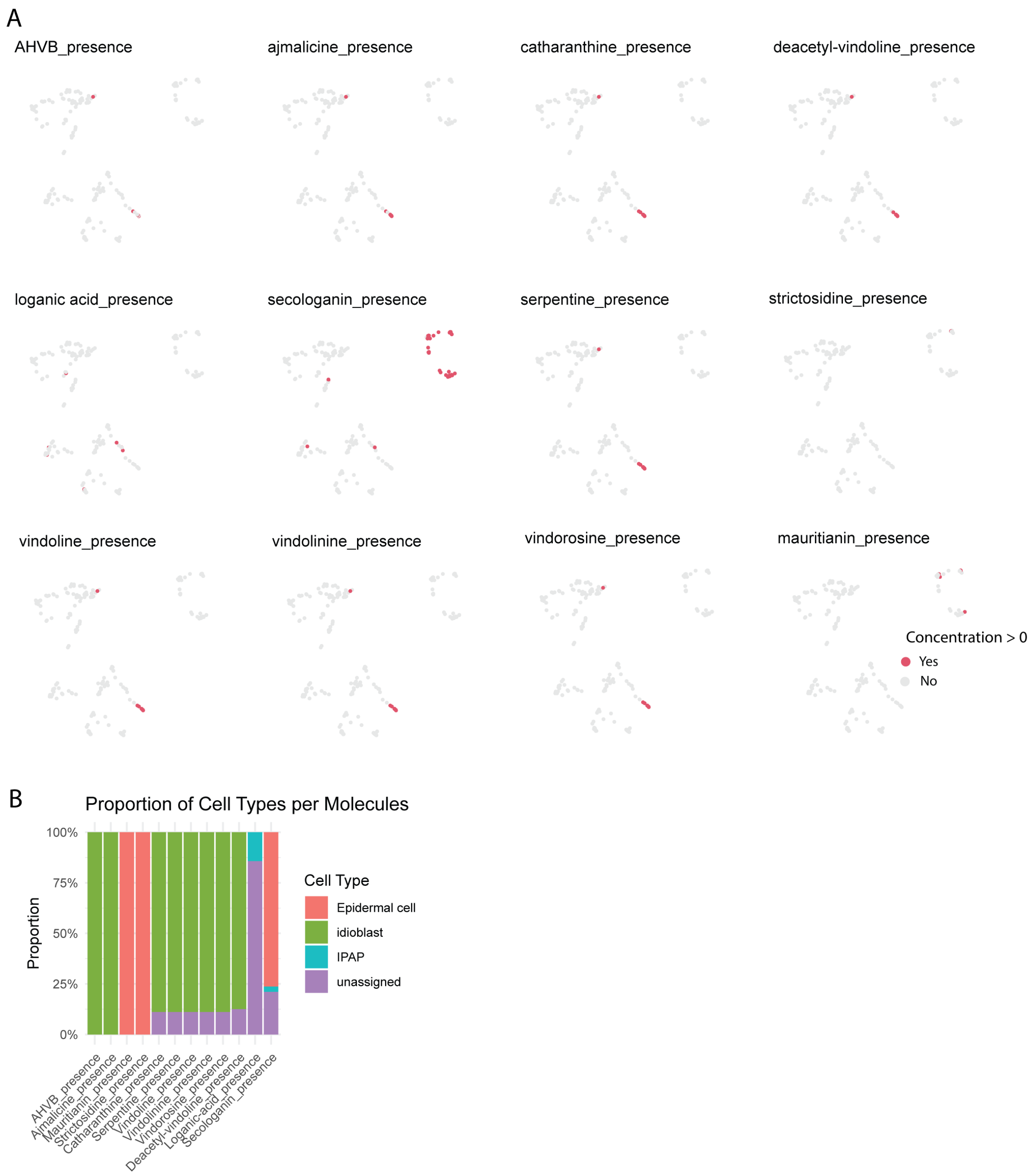

Fig. S17. Presence of targeted analytes in each cell type.

A. Presence of each analyte are plotted as pink dots on RNA-UMAP, when the peak area of each molecule is within quantifiable range. **B.** The stacked bar plot shows proportion of cells that retain each analyte. Cell types are colored based on their RNA-based cell type annotation.

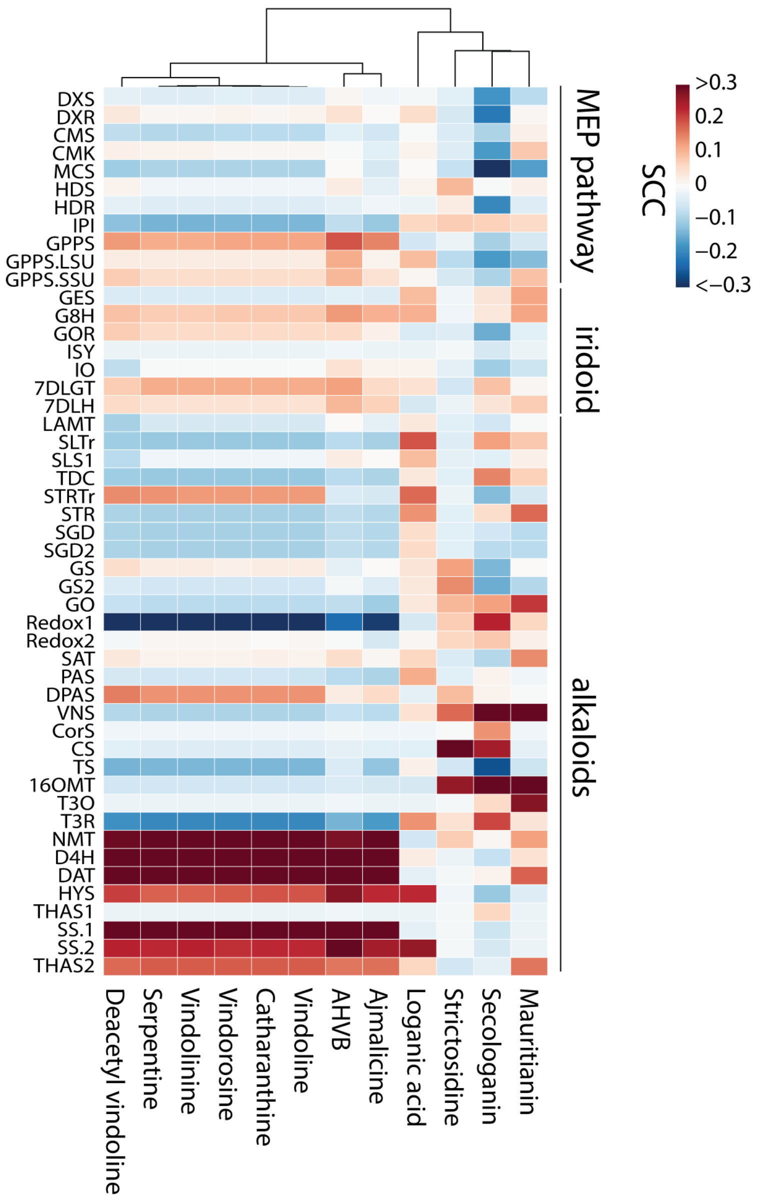

Fig. S18. Heatmap showing correlation coefficients of gene and metabolites.

Correlation between analytes and known biosynthetic genes are colored based on Spearman correlation coefficients. Late alkaloid genes such as CS, VNS show negative correlation with their products such as Catharanthine Vindoline, suggesting the molecules are actively transported in inter-cellular manner.

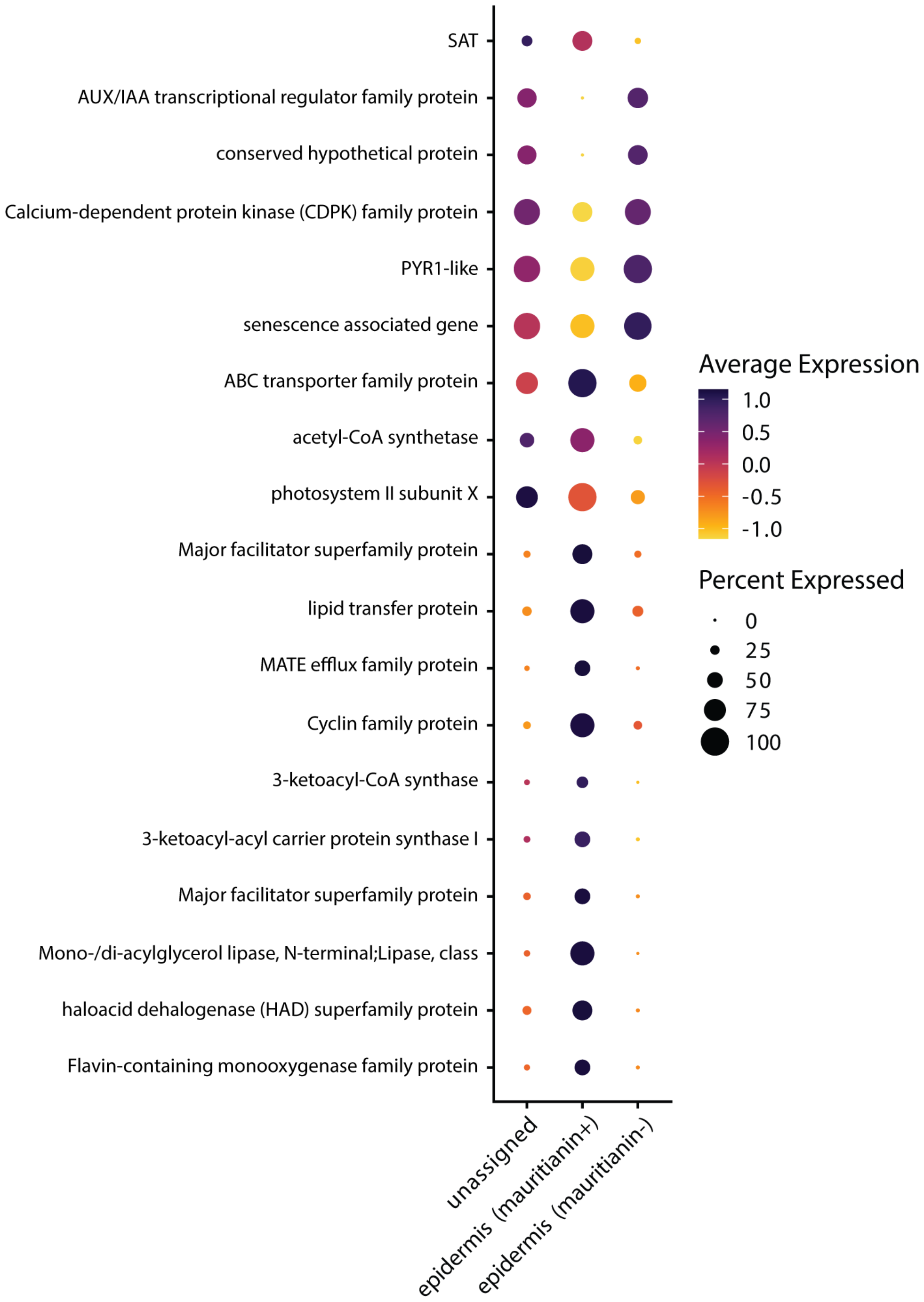

Fig. S19. Expression pattern of representative genes that are expressed differentially between two epidermis clusters that are accumulating different analytes**.** The dotplot shows that epidermal cells that accumulate a quantifiable amount of mauritianin are more likely to be active in lipid metabolism, whereas the other express more senescence or signaling cascade related genes.

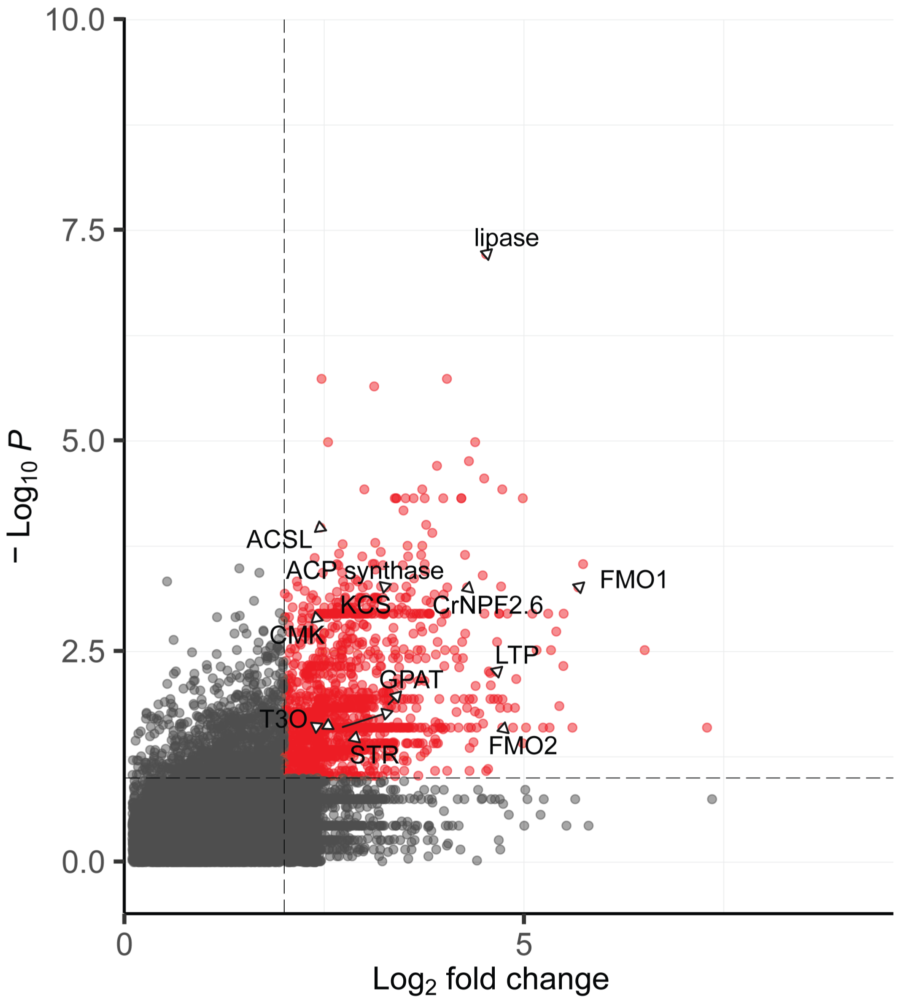

Fig. S20. Upregulated genes in epidermal cells that retain quantifiable levels of mauritianin.

Scatter plot showing upregulated genes in epidermal cells accumulating a quantifiable amount of mauritianin compared to those without a quantifiable level of mauritianin. Genes with $\log_2$FC > 2 and -$\log_{10}P$ value are shown in red. ACS synthase, 3-ketoacyl-acyl carrier protein synthase; ACSL, long-chain acyl-CoA synthetase; CMK, 4-(cytidine 5'-phospho)-2-C-methyl-D-erithritol kinase; FMO, Flavin-containing monooxygenase; GPAT, glycerol-3-phosphate acyltransferase; KCS, 3-ketoacyl-CoA synthase; lipase, Mono-/di-acylglycerol lipase; LTP, lipid transfer protein; STR, strictosidine synthase; T3O, tabersonine 3-oxygenase

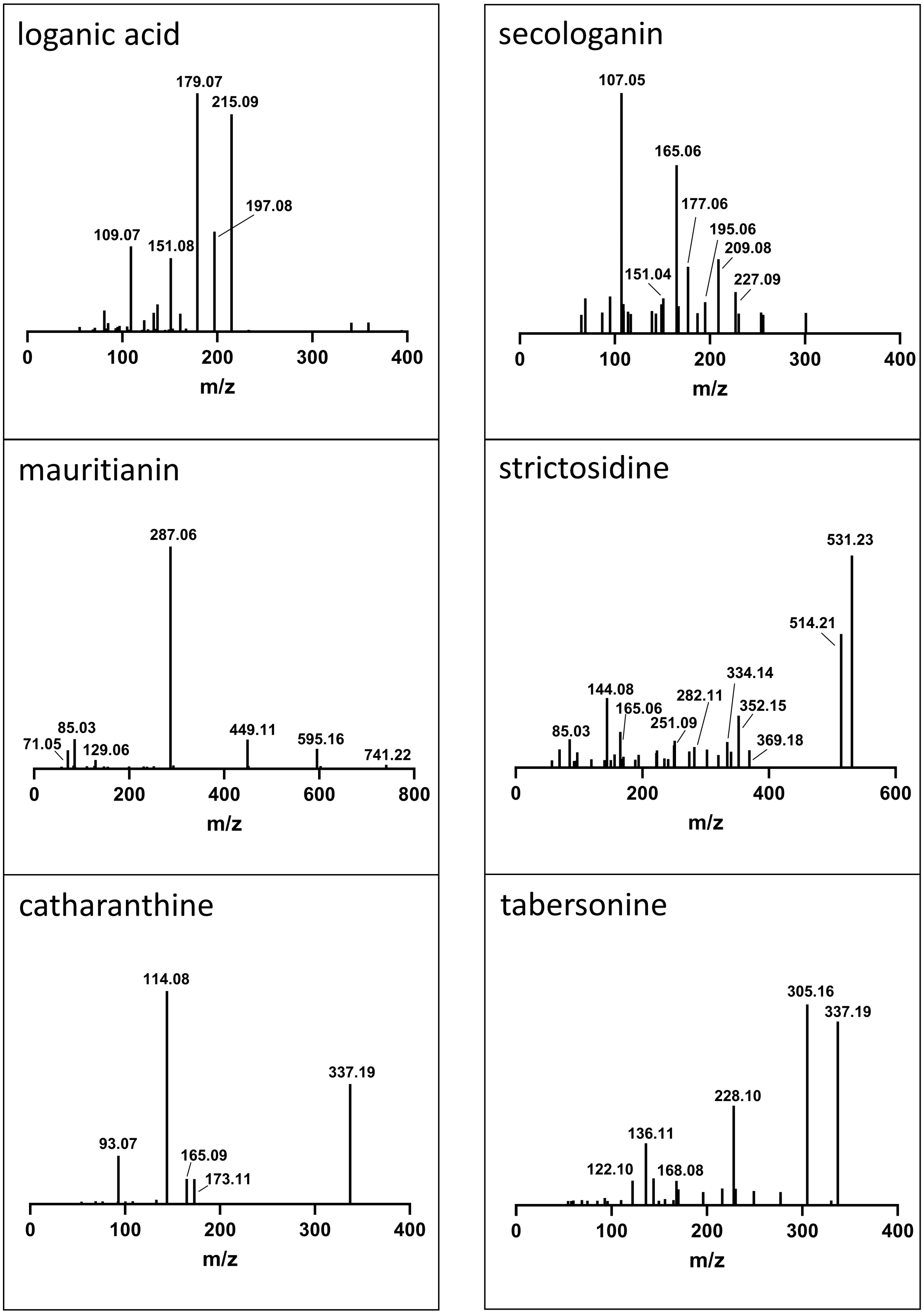

Fig. S21

Continued on next page →

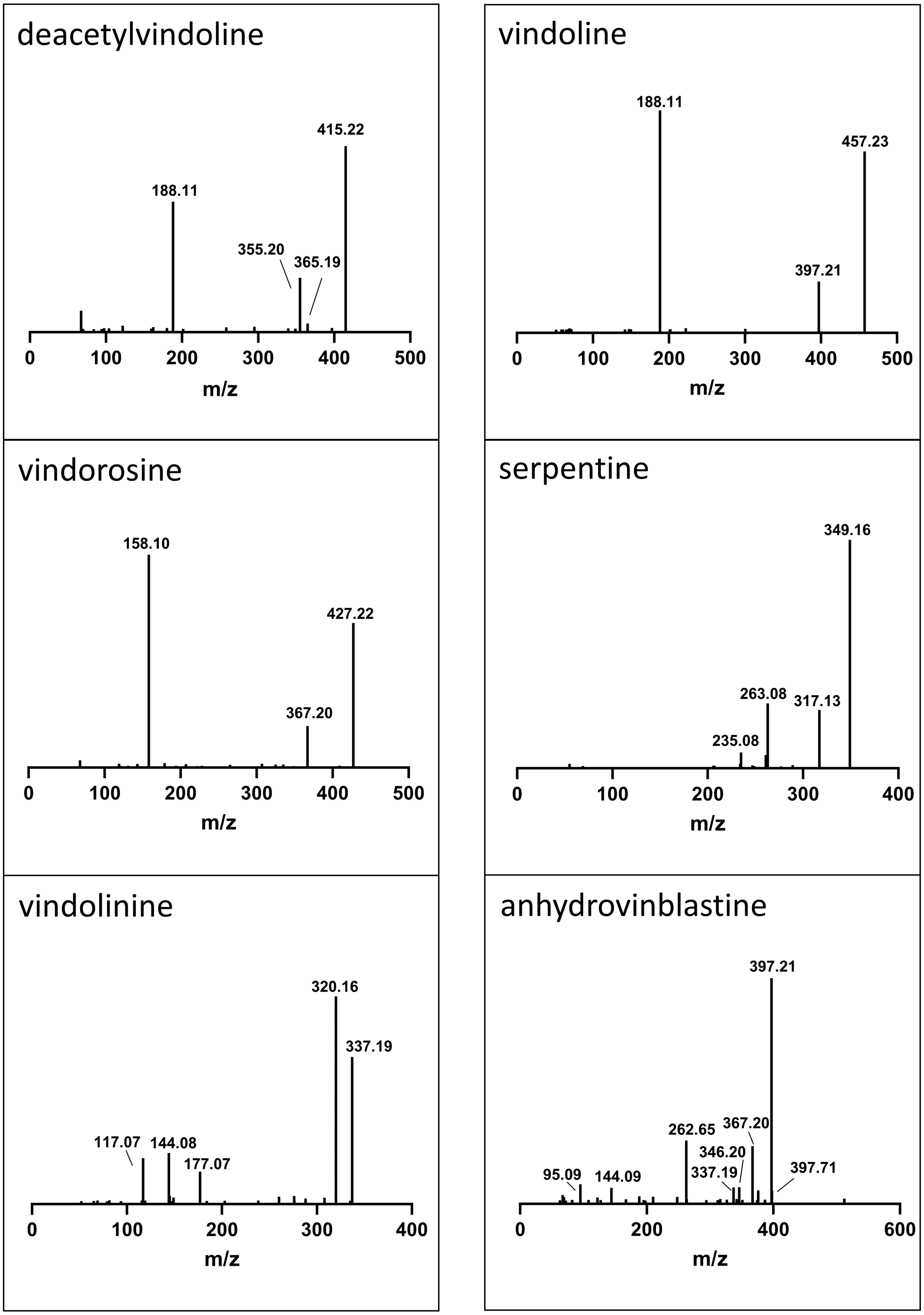

Fig. S21

Continued on next page →

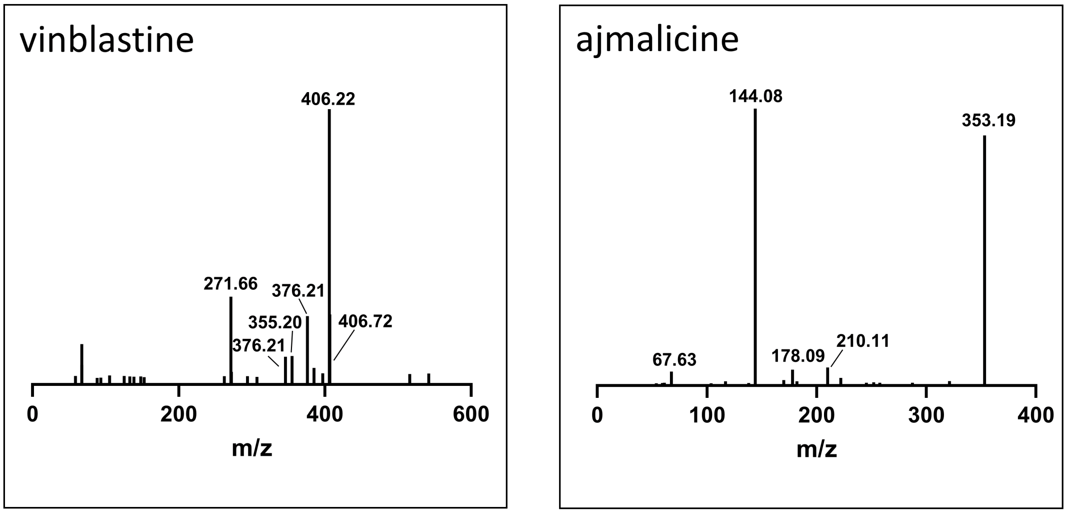

Fig. S21. MS^2 Spectra of compounds subjected to targeted analysis.

Table S1. Compounds used for identification.

Compound	Chemical formula	Ion	Measured m/z	Retention time (min)
loganic acid	$C_{16}H_{24}O_{10}$	$[M+NH_4]^+$	394.17041	1.43
secologanin	$C_{17}H_{24}O_{10}$	$[M+H]^+$	389.14377	2.00
mauritianin	$C_{33}H_{40}O_{19}$	$[M+H]^+$	741.22253	2.06
strictosidine	$C_{27}H_{34}N_2O_9$	$[M+H]^+$	531.23389	2.40
catharanthine	$C_{21}H_{24}N_2O_2$	$[M+H]^+$	337.19064	2.62
tabersonine	$C_{21}H_{24}N_2O_2$	$[M+H]^+$	337.19046	2.73
deacetylvindoline	$C_{23}H_{30}N_2O_5$	$[M+H]^+$	415.22247	2.55
vindoline	$C_{25}H_{32}N_2O_6$	$[M+H]^+$	457.23325	2.90
vindorosine	$C_{24}H_{30}N_2O_5$	$[M+H]^+$	427.22241	2.92
serpentine	$C_{21}H_{20}N_2O_3$	$[M]^+$	349.15448	2.63
vindolinine	$C_{21}H_{24}N_2O_2$	$[M+H]^+$	337.19077	2.13
anhydrovinblastine	$C_{46}H_{56}N_4O_8$	$[M+2H]^{2+}$	397.21222	3.10
vinblastine	$C_{46}H_{58}N_4O_9$	$[M+2H]^{2+}$	406.21692	2.88
ajmalicine	$C_{21}H_{24}N_2O_3$	$[M+H]^+$	353.18546	2.55

Table S2. Analytical parameters for the external calibration of compounds quantified in this study.

Compound	Calibration range (nM)	Regression equation[a]	Correlation Coefficient (R^2)	LOQ[b] (nM)
loganic acid	2-2000	y=12248x + 6150	0.9999	2
secologanin	10-1000	y = 16165x + 84261	0.9988	10
mauritianin	10-3000	y = 13958x + 42965	0.9998	10
strictosidine	0.1-500	y = 855571x + 379613	0.9999	0.1
catharanthine	0.1-400	y = 1039954x + 283600	0.9997	0.1
deacetylvindoline	0.05-500	y = 750146x + 1400257	0.9985	0.05
vindoline	0.1-500	y = 1186763x + 1795458	0.9991	0.1
vindorosine	0.1-500	y= 846541x + 597335	0.9996	0.1
serpentine	0.5-500	y = 1196468x + 269956	0.9994	0.5
vindolinine	0.05-150	y=2210449x + 710425	0.9993	0.05
anhydrovinblastine	0.02-50	y = 1976113x + 64437	0.9999	0.02
ajmalicine	0.05-200	y=1013368x+535542	0.9993	0.05

[a]Each point of calibration curve was measured in triplicate.

[b]The LOQ was estimated in the lowest analyte concentration injected that yielded a signal-to-noise (S/N) ratio of ≥10 in three replicates.

Table S3. Quantification of targeted analytes by scMS and their occurrence in cells after removing cells with unsuccessful scRNA seq analysis

Compounds	In this study			Vu et al., *JACS*, 2024 (1)			Li et al., *Nat. Chem. Bio* (2023)(2)		
	Mean (mM)	Min-max (mM)	No. cells detected/ No. cells analyzed (%)	Mean (mM)	Min-max (mM)	No. cells detected/ No. cells analyzed (%)	Mean (mM)	Min-max (mM)	No. cells detected/ No. cells analyzed (%)
anhydrovinblastine	0.34	0.12 - 0.71	5/193 (2.6)	2.62	0.31 - 11.8	7/202 (3.5)	0.3	0.01 - 1.4	37/627 (5.7)
ajmalicine	2.76	0.39 - 8.64	7/193 (3.6)	-	-	-	-	-	-
catharanthine	169.35	59.27 - 341.05	9/193 (4.7)	99.81	72.69 - 126.07	5/202 (2.5)	90.0	1.92 - 266.89	47/627 (7.5)
deacetylvindoline	1.07	0.39 - 1.62	8/193 (4.1)	0.36	0.19 - 0.53	5/202 (2.5)	-	-	-
loganic acid	49.21	5.63 - 218.37	7/193 (3.6)	7.32	1.5 - 49.26	36/202 (17.8)	-	-	-
secologanin	448.48	68.09 - 908.09	37/193 (19.2)	316.92	50.96 - 601.07	58/202 (28.7)	-	-	-
serpentine	16.50	8.47 - 35.66	9/193 (4.7)	4.91	2.44 - 7.35	5/202 (2.5)	18.3	2.25 - 43.98	47/627 (7.5)
stemmadenine acetate	-	-	-	-	-	-	-	-	-
strictosidine	0.76	-	1/193 (0.5)	-	-	-	-	-	-
vinblastine	-	-	-	0.10	-	1/202 (0.5)	0.2	0.08 - 0.52	5/627 (0.8)
vindoline	81.58	16.85 - 162.99	9/193 (4.7)	53.39	32.2 - 80.45	5/202 (2.5)	6.8	0.04 - 39.3	47/627 (7.5)
vindolinine	6.43	2.5 - 15.87	9/193 (4.7)	8.80	3.4 - 14.75	5/202 (2.5)	-	-	-
vindorosine	37.69	4.17 - 96.3	9/193 (4.7)	38.27	24.96 - 56.32	5/202 (2.5)	-	-	-
mauritianin	69.19	52.71 - 90.68	5/193 (2.6)	21.65	4.92 - 47.73	16/202 (7.9)	-	-	-
coronaridine	-	-	-	0.62	0.17 - 2.19	5/202 (2.5)	-	-	-

Table S4. Number of cells that were annotated as each cell type

Cell types based on RNA (UMAP plot of Figure 2A) were annotated based on known marker gene expression (*NLTP2* for epidermis, *G8H* or *ISY* for IPAP, and *D4H* or *DAT* for idioblast). MET based annotation was done as previously described in A. H. Vu *et al*. 2024, where secologanin served as marker for epidermis, serpentine did for idioblast, and loganic acid did for IPAP.

	RNA based annotation		MET based annotation	
**Cell type**	**Rep 1**	**Rep 2**	**Rep 1**	**Rep 2**
epidermis	14	16	15	23
idioblast	6	4	4	5
IPAP	4	4	4	3
parenchyma/unassigned	50	95	51	88

Table S5. Compound Discoverer™ parameters.

Nodes	Parameters		
Select Spectra	Scan Event Filters	Polarity Mode	Is +
Align Retention Times	General Settings	Alignment Model	Adaptive curve
		Maximum Shift (min)	0.5
		Mass Tolerance	5 ppm
Detect Compounds	Mass Tolerance	Mass Tolerance	5 ppm
		Intensity Tolerance	30
		Min Peak Intensity	10000
Group Compounds	Compound Consolidation	Mass Tolerance	5 ppm
		RT Tolerance	0.2 min
Fill Gaps	General Settings	Mass Tolerance	5 ppm
		S/N Threshold	1.5
Apply QC Correction	General Settings	Min. QC Coverage (%)	25
		Max. QC Area RSD (%)	80
		Max. Corrected QC Area RSD (%)	50
Mark Background Compounds	General Settings	Max. Sample/Blank	5
		Max. Blank/Sample	0
		Hide Background	True
Assign Compound Annotations	General Settings	Mass Tolerance	2 ppm
	Data Sources	Data Source#1	Predicted Compositions
	Scoring Rules	Use mzLogic	True
		Use Spectral Distance	True
		SFit Threshold	20
		SFit Range	20
Predict Compositions	Prediction Settings	Mass Tolerance	2 ppm
		Min. RDBE	0
		Max. RDBE	40
		Min. H/C	0.1
		Max. H/C	4
		Max. #Candidates	10
	Pattern Matching	Intensity Tolerance(%)	30
		Intensity Threshold (%)	0.1
		S/N Threshold	3
		Use Dynamic Recalibration	True
	Fragments matching	Use Fragments Matching	True
		Mass Tolerance	5 ppm
		S/N Threshold	3

SI References

1. A. H. Vu *et al.*, Quantitative Single-Cell Mass Spectrometry Provides a Highly Resolved Analysis of Natural Product Biosynthesis Partitioning in Plants. *J Am Chem Soc* **146**, 23891-23900 (2024).
2. C. Li *et al.*, Single-cell multi-omics in the medicinal plant Catharanthus roseus. *Nat Chem Biol* **19**, 1031-1041 (2023).
